# Phosphoproteome Analysis Reveals Phosphorylation Underpinnings in the Brains of Nurse and Forager Honeybees (*Apis mellifera*)

**DOI:** 10.1038/s41598-017-02192-3

**Published:** 2017-05-16

**Authors:** Gebreamlak Bezabih, Han Cheng, Bin Han, Mao Feng, Yu Xue, Han Hu, Jianke Li

**Affiliations:** 1 Institute of Apicultural Research/Key Laboratory of Pollinating Insect Biology, Ministry of Agriculture, Chinese Academy of Agricultural Science, Beijing, 100093 China; 2 School of Life Sciences, Zhengzhou University, Zhengzhou, Henan 450001 China; 3 Department of Bioinformatics & Systems Biology, College of Life Science and Technology, Huazhong University of Science and Technology, Wuhan, Hubei 430074 China

## Abstract

The honeybee brain is a central organ in regulating wide ranges of honeybee biology, including life transition from nurse to forager bees. Knowledge is still lacking on how protein phosphorylation governs the neural activity to drive the age-specific labor division. The cerebral phosphoproteome of nurse and forager honeybees was characterized using Ti^4+^-IMAC phosphopeptide enrichment mass-spectrometry-based proteomics and protein kinases (PKs) were predicted. There were 3,077 phosphosites residing on 3,234 phosphopeptides from 1004 phosphoproteins in the nurse bees. For foragers the numbers were 3,056, 3,110, and 958, respectively. Notably, among the total 231 PKs in honeybee proteome, 179 novel PKs were predicted in the honeybee brain, of which 88 were experimentally identified. Proteins involved in wide scenarios of pathways were phosphorylated depending on age: glycolysis/gluconeogenesis, AGE/RAGE and phosphorylation in nurse bees and metal ion transport, ATP metabolic process and phototransduction in forager bees. These observations suggest that phosphorylation is vital to the tuning of protein activity to regulate cerebral function according to the biological duties as nursing and foraging bees. The data provides valuable information on phosphorylation signaling in the honeybee brain and potentially useful resource to understand the signaling mechanism in honeybee neurobiology and in other social insects as well.

## Introduction

A typical honeybee (*Apis mellifera*) colony consists of three castes: the males, reproductive females and sterile workers^1, 2^. This division of labor further occurs in worker bees that perform different tasks according to age. The younger workers are mainly involved in in-hive activities as nurse bees before ultimately making the transition to forager bees that engage in nectar and pollen collection. This temporal behavioral development, known as age polytheism^3, 4^, is important for social organization. Normally, the nurse bees spend the first two to three weeks of adult life^5^ within the hive performing a wide range of tasks such as feeding young larvae, feeding the queen and performing hive maintenance^1^. The nurse bees also feed the younger and older bees^5^. They form a retinue around the queen to regulate queen behavior via the amount of royal jelly they feed to her, and act in spreading the queen’s pheromones across the nest^6, 7^. In contrast, once nurse bees become foragers, their duties move to a life stage dominated by foraging nectar, pollen, and colony defense^8^. This life transition is characterized by the distinctly different task performance of nurse and forager bees and by pronounced internal and physiological changes^9^. These changes are reflected in associative learning^10^, memorizing^11^, recognition^12^ and communication language with the hive mates^13^. To achieve the biological transition from nurses to foragers, the honeybees have an efficient central nervous system that can coordinate the complex social and behavioral interactions within the colony^9^. Therefore, the honeybee brain is a critical role player in the regulation of honeybee society by performing the cognitive, decision making, and communicative tasks during the transition from the nurse to forager stages of life^14, 15^. Despite being smaller than one cubic millimeter, the honeybee brain has about one million nerve cells, representing about one hundred-thousandth of the human brain^16^. The nerve cells in honeybee brains have the power to sufficiently perform various signals of indexical, iconic, and symbolic communication codes^17^. Honeybees efficiently regulate highly advanced social behaviors and intelligent decisions^16, 18^ by the functionality of brain cell chemistry, structure, endocrine activity, and changes in temporal patterns of gene and protein expression^19, 20^. For instance, juvenile hormone^4^, biogenic amines, dopamine, serotonin, and octopamine, play key roles in the brain with regard to the regulation of honeybee behavioral development^21^. Furthermore, neuro-molecules such as neuropeptides in the nerve cells function as neuromodulators, neurohormones, and neurotransmitters^21^ and have a major impact on peptidergic modulation of neural functions in bee brains^17^.

The nurse and forager phases are fundamentally important for colony organization and the physiological specialization during these two life stages is driven by variable protein expression in the brain of nurse and forager bees^22^. Protein phosphorylation, the most common post-translational modification (PTM), is a key switch for the rapid on-off control of signaling cascades that regulate cell differentiation and development, enzyme activity and metabolic maintenance in living cells^23, 24^. A fundamental mechanism for regulating signaling network and protein activity is the covalent PTM of serine (Ser), threonine (Thr), and tyrosine (Tyr) residues with phosphate^22, 24^. Given the advances in phosphopeptide enrichment and improvements in mass spectrometry (MS) instrumentation and methods, phosphoproteomics has enabled large-scale identification of protein phosphorylation sites and phosphorylation networks in biological samples. Although the proteome has been established in the brain of nurse and forager bees^22^, knowledge on how protein phosphorylation regulates age-specific neural activity in the honeybee brain is still lacking. Phosphoproteome analysis during the development of brood and salivary glands has been reported^25, 26^, but only very limited proteins were phosphorylated and phosphosites of those phosphoproteins were not discovered. Recently, an in-depth phosphoproteomic analysis of the hypopharyngeal glands of the honeybees revealed that dynamic protein phosphorylation networks tune the protein function to prime the gland development and functionality^11^. Therefore, the objective of this work is to provide a comprehensive characterization of phosphoproteome in the brains of nurse and forager bees that can potentially be useful to understand the phosphorylation events underlining age-specific cerebral functions on the basis of physiology.

## Experimental procedures

### Reagents

All chemicals were purchased from Sigma-Aldrich (St. Louis., MO, USA), otherwise the source was specified. Ti^4+^-IMAC material was bought from Dalian Institute of Chemical Physics, Chinese Academy of Sciences.

### Brain dissection and protein sample preparation

Honeybee (*Apis mellifera ligustica*) colonies used for sampling were raised at the apiary of the Institute of Apicultural Research, Chinese Academy of Agricultural Science, Beijing. The nurse and forager bees were sampled in accordance with the methods described by our group^27^. In short, newly emerged (<12 h after emergence) worker bees were marked on their thoraxes and placed back into the colonies to develop. The marked bees were collected as nurse bees after about days 10 with head extension to brood cells and as forager bees on day 20 at the entrance of the hive with a pollen load in the rear leg basket. There were 150 bees sampled from each of the five colonies headed by sister queens of the same age. Then, the brains were dissected as a pool sample and immediately stored at −80 °C for further analysis, and three independent biological replicates were produced per each treatment. All the colonies were managed with almost identical population, food, and brood during the nectar flow of chaste berry (*Vitexnegundo* L.).

Prior to protein extraction the brain tissue was homogenized on ice by pestle. The sample was then mixed with a lysis buffer containing 8 M urea, 2 M thiourea, 4% 3-[(3-cholamidopropyl) dimethylammonio]-1-propanesulfonate (CHAPS), 20 mM Trisbase, 30 mM dithiothreitol (DTT), 2% Bio-lyte (pH 3–10, and protease and phosphatase inhibitors (Roche, Basel, Switzerland)). The sample was centrifuged at 15,000 g at 4 °C for 15 min to remove the insoluble fractions. Ice-cold acetone was added to the recovered supernatant at −20 °C for 30 min to precipitate the proteins, then centrifuged twice at 15,000 g at 4 °C for 10 min. The protein pellets were dissolved in 40 mM (NH_4_) HCO_3_, then reduced with DTT (final concentration 10 mM) for 1 h to prevent reformation of disulfide bonds, and lastly, alkylated with iodoacetamide (final concentration 50 mM) for 1 h in the dark. Afterwards, sequencing grade modified trypsin (Promega, Medison, WI) was used to digest the protein (enzyme/protein ratio is 1:100 (W/W) sample at 37 °C for 14 hours.

### Phosphopeptide enrichment using Ti^4+^-IMAC and LC-MS/MS analysis

To enrich the phosphopeptides in the brains of nurse and forager worker bees, a high efficiency Ti^4+^-IMAC material was applied as previously described^28^. Specifically, the immobilized Ti^4+^ polymer beads (Ti^4+^-IMAC) were prepared by overnight incubation of 10 mg of polymer beads in 100 mM Ti(SO_4_)_2_ solution at room temperature (RT) under gentle stirring. The obtained Ti^4+^-IMAC beads were centrifuged at 20,000 g for 2 min. After removal of the residual titanium ions in the supernatant, distilled water was used to wash the Ti^4+^-IMAC beads. Before using those for the next step, the obtained Ti^4+^-IMAC beads were dispersed in 30% acetonitrile (CAN) containing 0.1% trifluoroacetic (TFA). Then, digested proteins were reconstituted in 500 μL of binding solution containing 6.0% TFA/80%ACN and incubated with 5 mg of Ti^4+^-IMAC material at RT for 60 min. The mixture was centrifuged at 13,500 g at 4 °C for 5 min. The supernatant was discarded and the precipitate was then washed with 200 μL of binding solution, with the washing buffer containing 0.6% TFA/50% ACN/200 mM NaCl, and 0.1% TFA/30% ACN. Thereafter, the bound phosphopeptides were eluted twice with 100 μL of 10% ammonia solution with vibration for 10 min. Finally, the enriched phosphopeptides were manually loaded onto Reversed-Phase Zip-Tip C18 columns (desalting column) for concentrating and desalting. The desalted peptides were extracted in a Speed-vac system (RVC 2–18, Marin Christ, Germany) and dissolved in 0.1% formic acid (FA); the extracted samples were stored at −80 °C for further LC−MS/MS analysis.

A sample of 8 µl of phosphopeptide per 0.5 µg specific amount of peptides with three technical replicate for each sample was loaded onto a Q-Exactive mass spectrometer (Thermo Fisher Scientific) and coupled to the EASY-nLC 1000 system using a nanoelectrospray ion source (Thermo Fisher Scientific). The samples were loaded onto a 2 cm long trap column (100 μm inner diameter fused silica containing 5.0 μm Aqua C18 beads, Thermo Fisher Scientific) for 2 min in buffer A (0.1% acetic acid) at a flow rate of 5 μL/min prior to separation. Then, the peptides were eluted from the trap column and subsequently separated in the analytical column (15 cm long, 75 μm inner diameter fused silica column filing with 3.0 μm Aqua C18 beads, Thermo Fisher Scientific). Peptides were gradient eluted in 180 min at a flow rate of 350 nL/min under the following conditions: from 5% to 8% buffer B in 5 min, from 8 to 20% buffer B in 115 min, then from 20 to 30% buffer B in 40 min, followed by an increase to 90% buffer B in 10 min and staying at 90% buffer B for an additional 10 min. The eluting peptides were directly infused into a Q-Exactive mass spectrometer (Thermo Fisher Scientific) via electrospray ionization **(**ESI). MS and MS/MS data were collected in a data-dependent mode using the following settings: one full scan (resolution 70,000 at m/z 400; m/z 300–1,800) followed by top 20 MS/MS scans using higher-energy collisional dissociation in the linear ion trap mass spectrometer (resolution: 17,500, isolation window: 2 m/z, normalized collision energy: 27) using dynamic exclusion (charge exclusion: unassigned 1, >8; peptide match: preferred; exclude isotopes: on; dynamic exclusion: 10 s). The MS/MS spectra of phosphopeptides were retrieved using Xcalibur (version 2.2, Thermo Fisher Scientific). The MS data have been deposited to the ProteomeXchange Consortium via the PRIDE (http://www.ebi.ac.uk) partner repository with the dataset identifier PXD003757.

### Database search, site localization and validation of phosphosites

The MS/MS data were processed and analyzed using in-house PEAKS software (version 8, Bioinformatics Solutions Inc.). A database containing protein sequences of *A. mellifera* (downloaded April, 2015 from NCBI) and common contaminants was integrated with a total of 21,778 entries. The search parameters were: trypsin specificity, fixed modification of carbamidomethyl (C)/+57.02 Da, variable modifications of oxidation (M)/+15.99 Da and phosphorylation (Ser, Thr, Tyr)/+79.96 Da, and two allowed missed cleavages per peptide; one non-specific cleavage at either end of the peptide; three maximum allowed variable PTM per peptide. Precursor mass tolerance was set at 15.0 ppm, and fragment ion tolerance at 0.05 Da. The false discovery rate (FDR) was controlled at both the protein and peptide levels using a fusion-decoy database search strategy at a threshold ≤1.0%, an enhanced target-decoy approach that makes more conservative FDR estimations^29^. Scaffold PTM (Version 2.1.3, Proteome Software, Oregon, USA) was used to assign the phosphosites by localization probability via Ascores algorithm^30^. All MS/MS of phosphopeptides queries with an Ascore for each site having a 95% or higher probability were considered. Abundance levels of phosphosites were quantified via spectral counting by summarizing the numbers of all peptide spectra of the phosphosite^31^.

To confirm the localized phosphosites on proteins in the nurse and forager brain, proteins with different abundance levels and peptides with different abundance levels in each protein were selected for validation. Eight selected phosphopeptides were commercially synthesized using a solid-phase peptide synthesis process (China Peptides Ltd. Co., Shanghai, China). The MS/MS spectra were compared between the digested phosphopeptides from the honeybee (*A. m. ligustica*) brain samples and the synthetic phosphopeptides. The phosphosites were considered to be validated only when the major ions in the spectra between the brain sample and the synthetic phosphopeptides were aligned (retention time shift tolerance <0.2 min and >90% b or y ions consistent).

### Motif analysis

Phosphorylation is catalyzed by protein kinases and these enzymes can be recognized by specific sequence motifs in theirsubstances^32^. The phosphorylation motif sets were extracted from all phosphopeptides with confident localized phosphosites (probability ≥95%) using a motif-X algorithm (http://motif-x.med.harvard.edu/motif-x.html)^33^. The background was the uploaded *A. mellifera* proteome (<10 M of database size that randomly generated from *A. mellifera* proteome), the motif width was 13, occurrences were 20, significance was 1*10^−6^, and motifs were extracted separately for Ser, Thr, and Tyr sites at position 7. The extracted motifs were used to determine the kinase classes (acidic, basic, proline-directed, tyrosine and others) based on substrate sequence specificity because the kinase specificity is often defined by amino acid motif surrounding Ser, Thr, and Tyr residues on the substrate proteins^23, 34^.

### Computational identification of site-specific kinase–substrate relations *(ssKSRs)* in nurse and forager bee brains

To identify ssKSRs in the honeybee brain, protein-protein interactions (PPIs) of *A. mellifera* were prepared by retrieving the database of STRING v10 (http://www.string-db.org/)^35^. Then, we mapped these proteins to the benchmark sequences of *A. mellifera* proteome (version 3.2) downloaded from Bee Base (www.beebase.org)^36^ by BLAST search. Finally, we obtained 906,294 non-redundant PPIs in 8,336 proteins of *A. mellifera*. Thereafter, group-based prediction system (GPS) software package, was used to predict the kinase-specific phosphosites^37^. As the developed GPS tool was mainly used for prediction of kinase-specific phosphosites in mammals and the protein kinases (PKs) of *A. mellifera* were not included in the GPS 2.1 program, we first identified 231 potential PKs in *A. mellifera* based on the Hidden Markov Model (HMM) profiles and Ortholog searches, and this model classified the PKs in a hierarchical structure composed by group, family, and single PKs^38^. Because GPS algorithm can only predict kinase-specific phosphosite at the PK cluster level, the links between the PKs of *A. mellifera* with their corresponding GPS 2.1 predictors, if available, were manually formed^39^. In total, there were 179 PKs with 55 GPS predictors that were identified in *A. mellifera* (Supplemental Table 5). Then, the exact PKs of the identified phosphosites were characterized. Furthermore, the integrated PPIs were adopted as filter to reduce potential false positive hits of the predicted ssKSRs. If the kinase–substrate relations (KSRs) were supported by PPIs, the predictions of GPS were reserved. During the prediction, all items of the phosphorylation site peptide (PSP) (15, 15) were extracted from the brain phosphoproteomes of nurse and forager bees, and the middle threshold was employed for GPS 2.1.

### Construction of kinase and substrate interaction network

A protein kinase can phosphorylate a protein at multiple phosphosites; this may cause more than one ssKSRs between the PK and substrate. For the construction of kinase-substrate phosphorylation network (KSPN), we only considered the KSR, while multiple ssKSRs of a PK and its substrate were regarded as a single KSR. For the predicted ssKSRs in the brain of nurse and forager bees, the KSPNs were constructed and visualized with the software Cytoscape 3.3. In the phosphorylation networks, the nodes represented PKs or substrates, whereas the edges were KSRs. Given that the KSPN is directional^39^, the orientations were defined as Kinase >Substrate (a PK phosphorylates a protein which is not a PK) and Kinase - >Kinase (a PK phosphorylates a protein which is also a PK).

### Quantification of phosphoprotein abundance levels and GO term enrichment

To evaluate the expression level of phosphoproteins in the brains of nurse and forager bees, raw MS data was processed in PEAKS Q module (version 8, Bioinformatics Solutions Inc.). Then, the changes in protein abundances levels of the brain across two ages in each of the nurse and forager bees were quantified. Peptide ion abundance in the three replicates was used to calculate the expression level of each protein. Based on an expectation-maximization algorithm, feature detection was employed separately on each sample^40^. Then, using a high-performance retention time algorithm, the features of the same peptide from different samples were reliably aligned^40^. Calculations of the protein *p*-value (one-way ANOVA) were then performed on the sum of the normalized abundances across all runs. ANOVA values of p ≤ 0.05 and regulation of ≥2 fold change were regarded as significant regulated proteins between the nurse and forager bees. ClueGO integrated with Gene Ontology (GO) and KEGG/BioCarta pathways is useful to create functionally organized GO/pathway network, and also important to compare/analyze two lists of genes and comprehensively visualizes functional grouped terms^41^. To provide in-depth knowledge with regard to the biological implications of the identified phosphoproteome in the brain of the honeybee, the identified phosphoproteins were used as an input for functional enrichment of GO term using ClueGOv2.1.6, a Cytoscape plug-in (http://www.ici.upmc.fr/cluego/)^41^. A right-sided hyper-geometric test was used to report the significantly enriched, functional GO categories in functional classes and pathways by comparing the input data with the background set of GO annotations in the honeybee genome. Based on their kappa score level (0.4) in ClueGO, the nodes in functionally grouped networks were linked. Functional categories and pathways were only considered significantly enriched when the *p*-value was <0.05. An FDR was controlled with a Bonferroni step-down test to correct the p-value of GO terms.

### Quantitative real-time PCR (qPCR)

To survey the differentially expressed proteins associated with brain functions in nurse and forager bees at the gene level, brain tissue was mixed by pestle homogenization. Total RNA was extracted from the brain samples of nurse and forager bees using TRIzol reagent (Invitrogen, USA) according to the manufacturer’s instructions and quantified with a NanoDrop ND-1000 spectrophotometer (NanoDrop Technologies). To test the quality and integrity of total RNA, the bands of 28S RNA, 18S RNA, and 5S RNA were visualized with 1.0% agarose gel electrophoresis. Then cDNAs were generated using reverse-transcriptase kit reagents (Transgen, China). From the differentially expressed proteins, nine highly abundant proteins (*Mob3*: Mps one binder kinase activator-like 3, *ACCB14939*: leucine-rich repeat serine/threonine-protein kinase, *Adk*: adenylate kinase, *Phl*: raf homolog serine/threonine-protein kinase phl, *LOC552007:* pyruvate kinase-like isoform X3*, CamkII*: calcium-independent protein kinase C, *PDPK1*: 3-phosphoinositide-dependent protein kinase 1, *CDK10:* cyclin-dependent kinase 10, and *LOC409276:* phosphatidylinositol 5-phosphate 4-kinase type-2 beta) were selected for qRT-PCR analysis. The specific primers used for qRT-PCR are provided in Supplemental Table 1. Glyceraldehyde-3-phosphate dehydrogenase (GAPDH) was used as a reference gene to normalize the data. PCR amplification and data collection were conducted by an iQ5Multicolor Real-Time PCR Detection System (Bio-Rad, Hercules, CA) according to our previous method^11^. The statistical analysis of gene expression was performed using an independent samples t-test (SPSS version 16.0, SPSS, Inc. Chicago, IL, USA). An error probability of p < 0.05 was considered statistically significant.

## Results

### Phosphoproteome Profiling of Nurse and Forager Honeybee Brains

In an effort to map the phosphoproteome in the brains of nurse and forager bees, high efficiency IMAC^+4^ phosphopeptide enrichment and state-of-the-art MS were employed. In all, 4,138 phosphosites resided on 4,192 non-redundant phosphopeptides derived from 1,244 phosphoproteins were identified with FDR < 1.0% at both peptide and protein levels (Supplemental Tables 2 and Table 3). There were 3,234 phosphopeptides from 1,004 phosphoproteins in the nurse bee and 3,110 phosphopeptides from 958 phosphoproteins in the forager bees (Supplemental Table 2 and 3). Of the 3,234 and 3,110 identified phosphopeptides, belonged to 4,192 non-redundant phosphopeptides, 1,082 were unique to nurse bees, 958 were unique to forager bees, and 2,152 were shared between them (Supplemental Fig. 1). Most of the phosphopeptides were phosphorylated on single sites (78.7%), followed by double (20.6%) and triple (0.7%) sites in nurse bees (Supplemental Fig. 2A). A similar ratio was also found in the forager bees for phosphorylation on single (81.9%), double (17.5%), and triple sites (0.6%) (Supplemental Fig. 2B). Of the total of 1,244 phosphoproteins, 286 were unique to nurse bees, 240 were unique to forager bees, and 718 were shared between them (Supplemental Fig. 3). Eight phosphopeptides from eight phosphoproteins with different dynamic ranges of abundance level were selected to validate the phosphorylation of the site on the peptides. The spectra of eight artificially synthesized phosphopeptides were compared with the spectra in the brain sample, the eight phosphosites were validated (Supplemental Fig. 4). The reported data is the first comprehensive phosphoproteome in the honeybee brains.

### Age-specific phosphorylation pattern in honeybee brain

**Figure 1:**
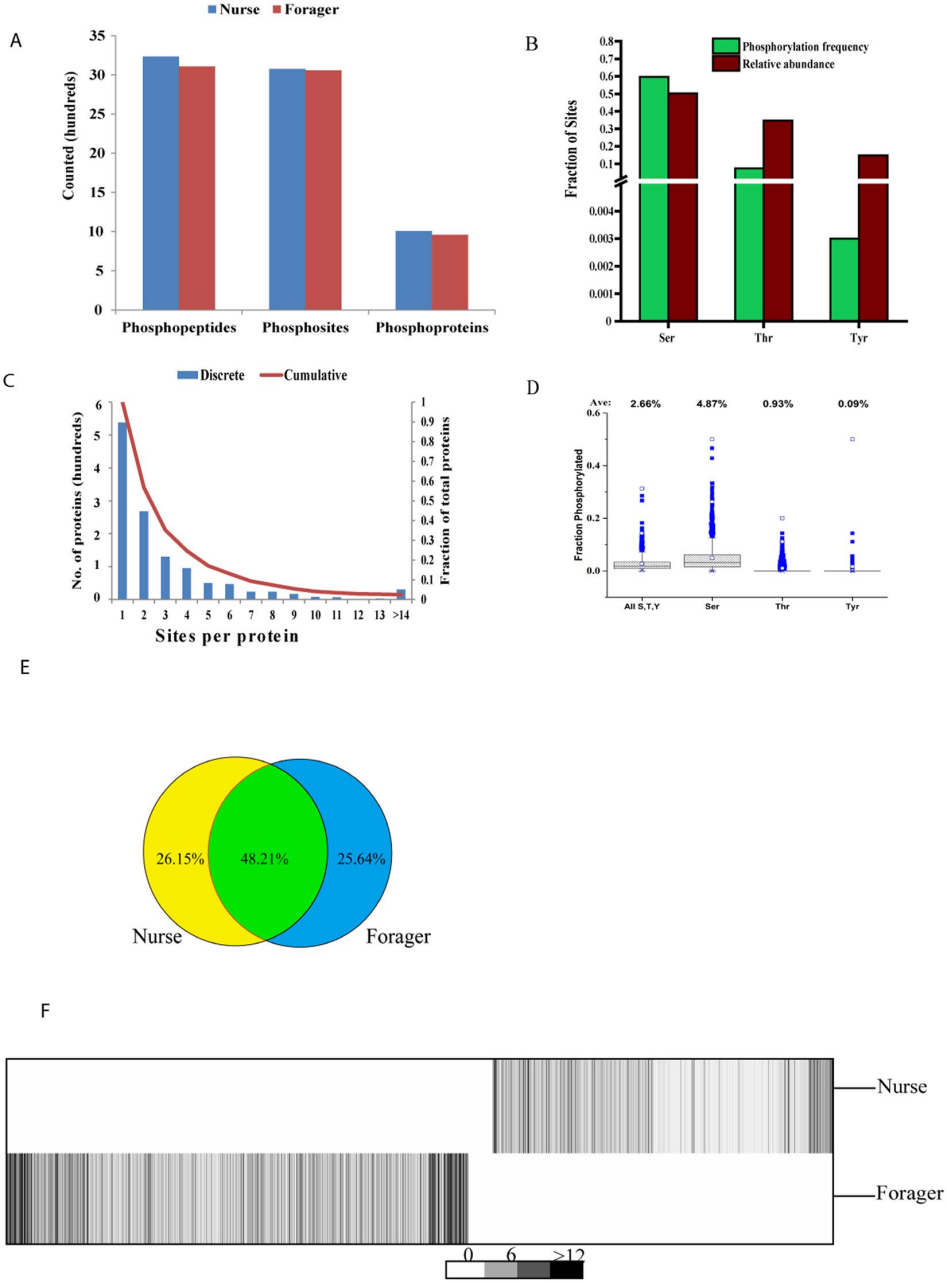
Overview of age-specific phosphorylation in the brain of honeybee (*A. m. ligustica*). (**A**) Number of phosphopeptides, phosphosites and phosphoproteins detected at distinct ages of the honeybee brain. (**B**) Relative frequencies of Ser, Thr, and Tyr within all phosphoproteins and their relative likelihood of phosphorylation. The relative abundances found in the data indicate the relative number of Ser, Thr, or Tyr (total-phosphorylation and non-phosphorylation) found in the data. (**C**) Histogram depicting numbers of sites observed per protein. (**D**) Box plots indicating the relative extents of modification for residues prone to phosphorylation within all observed phosphoproteins. (**E**) Pie chart indicating numbers of phosphosites detected in the nurse and forager honeybees. (**F**) Hierarchical clustering of phosphosites and different ages of the honeybee brain based on number of spectral counts. The two ages were compared and the sums of the average spectral count were 32,152 and 22,751for forager and nurse honeybees, respectively.

**Figure 2:**
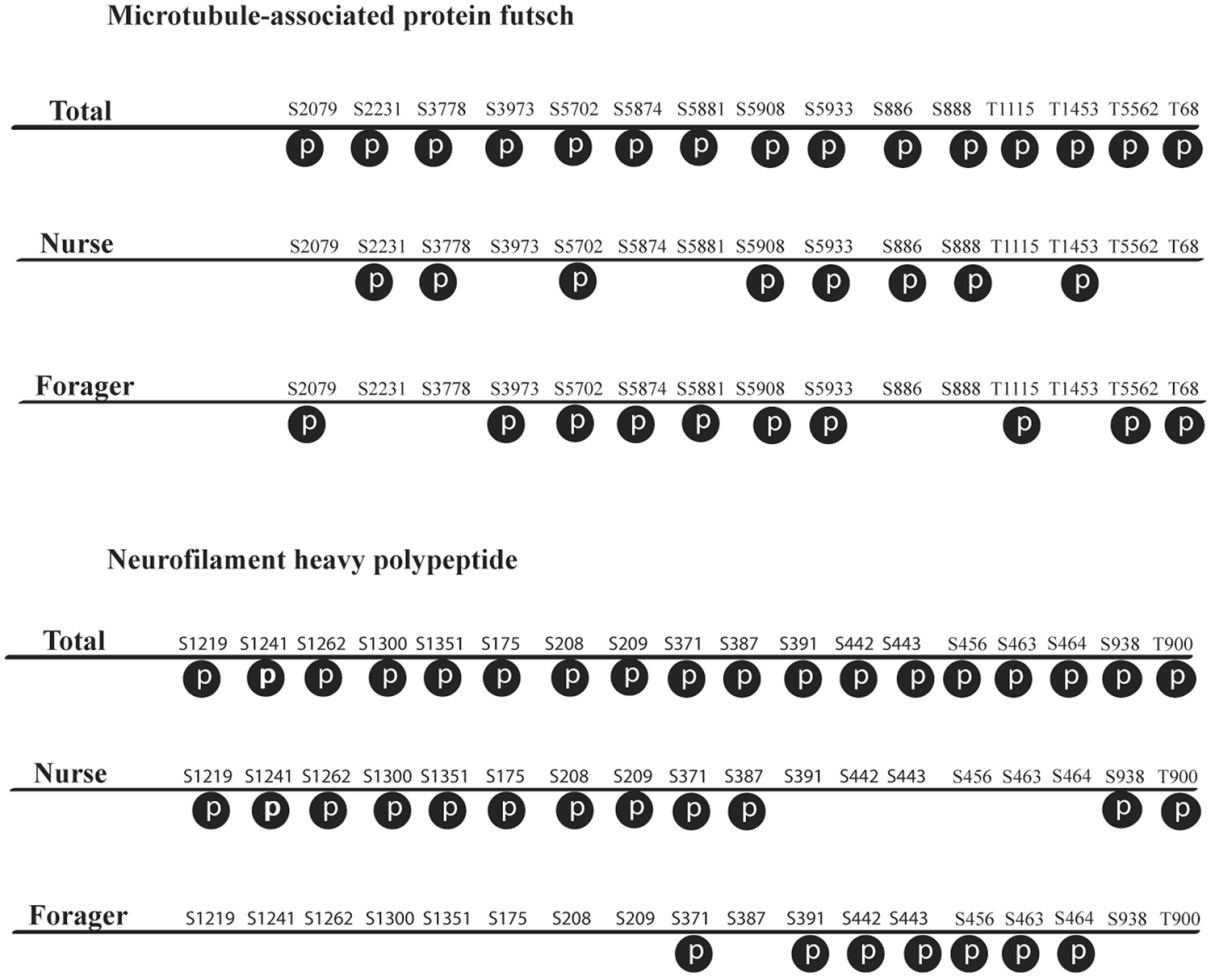
Phosphorylation patterns of two selected phosphoproteins identified in our dataset in the nurse and forager bees. (**A**) Microtubule-associated protein futsch. (**B**) Neurofilament heavy polypeptide. “S” stands for serine and “T” stands for threonine, the number indicates the position of the amino acid in the polypeptide chain, and phosphosite in depicted as a “P” in the black circle.

Phosphorylation is usually reflected at three levels, the number of phosphosites, phosphopeptides, and phosphoproteins. A higher number of phosphosites, phosphopeptides and phosphoproteins were identified in nurse bees than in forager bees (Fig. 1A). Also a high portion of phosphorylation of Ser (88.4%) was observed, followed by Thr (11.2%) and Tyr (0.4%). Phosphorylation of Ser was preferred to Thr and Tyr in the residues that were subjected to the phosphorylation within the phosphoproteins in the bee brain (Fig. 1B, Supplemental Fig. 5A and B). We then analysed the numbers of sites within each phosphoprotein. 43.3% of phosphoproteins contained single site, whereas 56.8% were phosphorylated on multiple residues of which 2.0% carried 14 or more sites (Fig. 1C). Specifically, 47.2% of the proteins were phosphorylated at a single site, 52.8% at multiple sites, and 2.6% at 11 or more sites in nurse bees. In forager bees, these numbers were 44.3%, 55.7%, and 3.1%, respectively (Supplemental Fig. 6A and B). Overall, 2.7% of the Ser, Thr and Tyr residues were modified, with some variability for each residue: Ser, 4.9%; Thr, 0.9%; Tyr 0.1% (Fig. 1D). Moreover, about 48.2% of the phosphosites were shared by nurse and forager bees, whereas 26.2% and 25.6% were unique for each of them (Fig. 1E). Specificaly, of all the Ser residues, 943 unique to nurse bees, 906 unique to forager bees and 1,816 were shared; of all the Thr residues, 134 unique to nurse bees, 151 unique to forager bees and 171 shared and of all the Tyr residues, 5 unique to nurse bees, 4 unique to forager bees and 8 shared were observed (Supplemental Fig. 7). Among the unique sites in the two different age groups, similar age-specific distributions were found in nurse bees (50.49%) and forager bees (49.5%). To better assess age-dependent distribution, the age specific phosphosites are shown in Table 1. These sites were derived from proteins with variable levels of abundance, such as serine/threonine-protein kinase BRSK2-like isoform X1 and major royal jelly protein 7 precursor exclusively found in forager bees. Protein 4.1 homolog isoform X8 and the splicing factor 1-like isoform X1 were found only in nurse bees. For comparison, the proteins commonly phosphorylated in nurse and forager bees are shown in Table 2. Examples of these proteins are elongation factor 1-beta’, hsp90 co-chaperone Cdc37 and neurofilament heavy polypeptide. Though some sites were phosphorylated in both nurses and foragers, extensive age-specific phosphorylation patterns were observed. Firstly, even if commonly expressed proteins displayed considerably different phosphorylation profiles across the two ages, the heavily phosphorylated microtubule-associated protein futsch (35 sites) harbored an abundant nurse-specific site (S2231 and T1453). Secondly, many proteins were phosphorylated at one specific age of the honeybee brain. For instance, the proteins only found in single ages are reticulon-4-like isoform X3 and protein 4.1 homolog isoform X8 (nurse), mediator of DNA damage checkpoint protein 1 isoform X1 and peripheral plasma membrane protein CASK-like isoform X1 (forager). To compare phosphorylation events for each site at two ages, the site abundance was hierarchically clustered using the total spectral counts of each site. An apparent difference in phosphorylation profiles for each site was found between nurse and forager bees (Fig. 1F). Generally, high abundance levels of phosphosites were observed in the forager bees than the nurse honeybees based on the spectral counts. Furthermore, a single protein carried several phosphosites and those phosphosites showing differential phosphorylation patterns were observed in both honeybee ages (Fig. 2).

**Table 1:** Abundant age-specific phosphosites in worker honey bee brain at two ages as determined by spectral counts.

Age	Annotation	Accession	Novel?^a^	Site	Class^b^	Nurse^c^	Forager^c^	Motif sequence
Nurse	protein 4.1 homolog isoform X8	gi|571525422	Y	S24	P	11	0	NGTDTNSPTKSPV
	uncharacterized protein DDB_G0284459-like	gi|328791406	Y	S25	P	19	0	ASFEDLSDEVENG
	splicing factor 1-like isoform X1	gi|571530871	Y	S47	P	31	0	PEERSPSPEPIYS
	voltage-dependent calcium channel type A subunit alpha-1 isoform X23	gi|571516489	Y	S847	P	34	0	VEICPPSPNQNFK
	probable G-protein coupled receptor B0563.6-like isoform X1	gi|571518442	Y	T381	P	4	0	GTTVRLTPEQTKL
	epidermal growth factor receptor pathway substrate clone 15	gi|571540760	Y	S694	P	124	0	APPRPESPSPALP
	reticulon-4-like isoform X3	gi|571549012	Y	S10	P	53		EPACPMSPGKEKE
	microtubule-associated protein futsch	gi|571524355	Y	S1130	A	41	0	QDVLNESFEGEEL
				S1473	A	18	0	SPEPAVSEIDLLE
	high mobility group protein DSP1, partial	gi|571530959	Y	T300	P	3	0	AEMQNYTPPKGES
Forager	serine/threonine-protein kinase BRSK2-like isoform X1	gi|328776379	Y	S328	P	0	4	LIQELLSPNHNTE
	collagen alpha-1(IV) chain-like isoform 1	gi|328782977	Y	S69	P	0	6	VTNNEGSDEADRR
	oxysterol-binding protein 1 isoform X3	gi|571538215	Y	S137	A	0	12	AIQAMESEEEEEE
	DNA ligase 1-like isoform X1	gi|571571344	Y	S33	P	0	9	LDFENESPKENGN
	major royal jelly protein 7 precursor	gi|62198227	Y	S426	A	0	20	NIQNDDSDENNDD
	disks large homolog 5-like isoform X6	gi|328789808	Y	S1532	P	0	4	EDQNRKSPPPSEP
Forager	uncharacterized protein LOC410819 isoform X2	gi|571515300	Y	T157	P	0	49	NPPLCLTPTHSGP
	mediator of DNA damage checkpoint protein 1 isoform X1	gi|571506305	Y	S872	P	0	24	MDSERDSPLPSNI
	peripheral plasma membrane protein CASK-like isoform X1	gi|571547686	Y	T422	P	0	8	ISTRVPTPCRAPQ
	short spindle protein 4 isoform X1	gi|571510304	Y	S505	P	0	16	IENFGGSQDNLHN

The data in this table chooses abundant phosphosites that were found in only one of nurse and forager bee brain, listed by protein and bee of different age. Age-specific sites are shown for the two ages of the brain in honeybee workers. ^a^Novel site is decided according whether the site has been reported with protein post-translational modifications in *Apis mellifera* or not. ^b^Site class is assigned by a decision tree algorithm: A = acidic; B = basic; O = other; P = proline-directed; T = tyrosine. ^c^Number of spectral count in a protein of nurse and forager bees.

**Table 2:** Abundant “Common” phosphosites in honeybee brain at the two ages as determined by spectral counts.

Annotation	Accession	Novel?^a^	Site	Class^b^	Nurse^c^	Forager^c^	Motif sequence
prostaglandin E synthase 3-like isoform X2	gi|571575529	Y	S157	A	116	238	WKDEDDSDDEGGM
microtubule-associated protein futsch-like	gi|571525698	Y	S1229	P	126	193	DSFEIESPPLVSP
uncharacterized protein LOC413428	gi|571563084	Y	S1473	O	117	185	RTESVDSDAGPDF
epidermal growth factor receptor pathway substrate clone 15	gi|571540760	Y	S694	P	124	165	APPRPESPSPALP
elongation factor 1-beta'	gi|66565249	Y	S99	A	40	104	DVDLFESDEEEDP
hsp90 co-chaperone Cdc37	gi|335892823	Y	S13	A	50	100	WKDIEISDDEDDT
uncharacterized protein DDB_G0284459-like	gi|328791406	Y	S865	P	69	84	TAGKSPSPPPLPP
neurofilament heavy polypeptide	gi|110755329	Y	S56	A	8	2	AAAAPASQEAAAA
microtubule-associated protein futsch	gi|571524355	Y	S1130	A	41	45	QDVLNESFEGEEL
28 kDa heat- and acid-stable phosphoprotein-like	gi|110762783	Y	S110	A	4	6	LSQLNQSLDSAKP
phosphatidylinositol-binding clathrin assembly protein LAP isoform X8	gi|571544367	Y	T301	P	16	8	KGSAANTPTQSAS
synaptotagmin 1	gi|224809489	Y	S152	P	102	66	SASMRDSKSSSQS
protein kinase C iota type isoformX1	gi|328777457	Y	T750	P	56	41	ECSPHNSPHDDIF
ATP-binding cassette sub-family A member 2-like isoform X2	gi|328778864	Y	T1626	P	24	22	LLGGDCTPTTPSG
phosphatidylinositol 5-phosphate 4-kinase type-2 beta-like isoformX1	gi|328785105	Y	S338	P	15	22	MTTPPESPHAALM
excitatory amino acid transporter 2 isoform X1	gi|571513382	Y	T516	P	55	65	EDLEMGTPLENIL
probable Ras GTPase-activating protein-like isoform X2	gi|571520333	Y	S1272	P	14	25	GVDLSPSPPPQNN
rabconnectin-3A	gi|571536236	Y	S1451	A	47	55	NLQHQESDEFQAS
autophagy 1	gi|66564768	Y	S197	A	16	33	IKDKWESFSEGEI
potassium voltage-gated channel subfamily H member 7-like isoform X6	gi|571570975	Y	S961	O	12	26	LPHAWHSQPAAFR

Selected abundant phosphosites that were found in the brain of nurse and forager bees (*Apis mellifera L*.), listed by protein. See also Supplemental Table 2 and 3. ^a^Novel site is decided according to whether the site has been reported with protein post-translational modifications in *Apis mellifera*. ^b^Site class is assigned by a decision tree algorithm: A = acidic; B = basic; O = other; P = proline-directed; T = tyrosine. ^c^Number of spectral counts in a protein of nurse and forager bees.

### Four kinase classes modify most phosphoproteins

**Figure 3:**
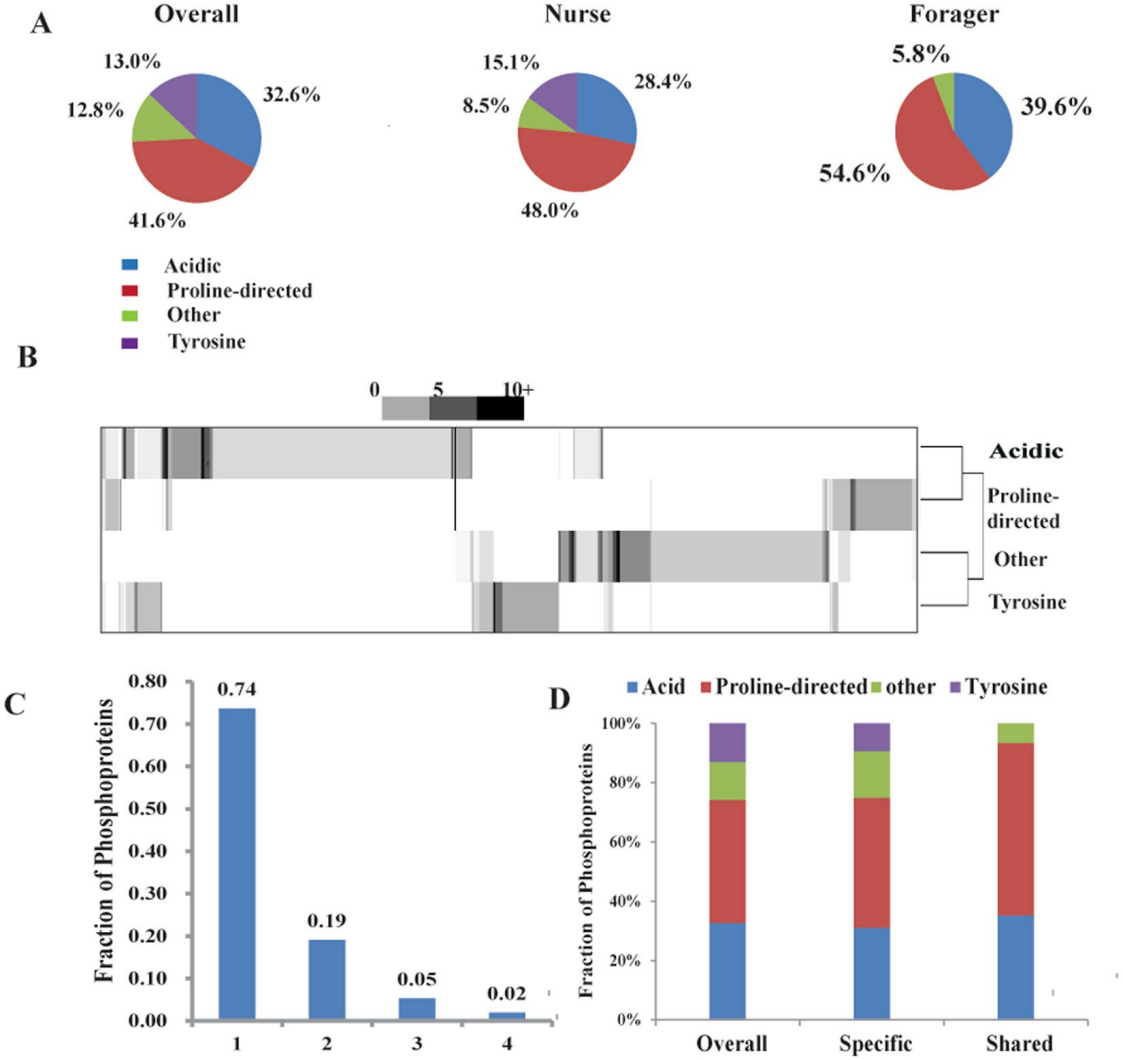
Overview of phosphosite classes across the different ages. (**A**) The relative frequencies with which each class is observed overall and for each worker age is plotted as pie charts. (**B**) The heat map presents the numbers of sites of each class observed for 566 phosphoproteins. Proteins and site classes have been clustered to highlight similarities. (**C**) Histogram indicating relative proportions of phosphoproteins containing phosphosites from variable numbers of classes. (**D**) Bar graph indicating relative proportions of age-specific and shared phosphosites in each.

Most protein kinases show phosphorylation motif specificity or at least phosphorylation motif preference^42^. Kinase motifs in different biological pathways often utilize the same general motif^31, 34^; and it can be divided into five classes: acidic, basic, proline-directed, tyrosine and “other” on the basis of a decision tree. To examine the kinase classes implicated in the neural activity during the phosphorylation in the honeybee brain, the amino acid motifs surrounding each site from the phosphoproteome data were extracted. On the basis of the extracted motifs, 4 kinase classes, proline-directed, acid, tyrosine and “other” were predicted (Supplemental Table 4). Acidic kinase motifs such as [S]-X-D and [S]-X-E and proline-directed kinase motifs [S]-P, [T]-P and [T]-PP as well as “other” kinase motifs such as [S]-X-P, [Y]-X-P and D-[S] were identified in both ages of bees. Based on the abundances of site classes of the motif, proline-directed sites were the dominant class (41.6% sites), followed by acidic (32.64% sites), tyrosine (13.0% site) and others (12.8% sites) (Fig. 3A). The nurse and forager bees showed distinct distributions between the kinase classes (Fig. 3D). In particular, the forager bees showed no tyrosine phosphorylation, whereas it was nearly 15% in nurse bees. We then analyzed the abundance of site classes within each phosphoprotein using hierarchical clustering, a quite distinct pattern was found by heat map representation (Fig. 3B). About 74% of the phosphoproteins contained a single site class, either proline-direct, acidic, “other” or tyrosine and 26% of phosphoproteins contained multiple sites classes, while 2% had sites from 4 site classes (Fig. 3C). Examples of the two variably phosphorylated proteins were spectrin beta chain isoform X6 and oxidation resistance protein 1-like isoform X1. Each was phosphorylated across the protein length and contained three kinase targeted site classes: proline-direct, acidic, and tyrosine (neither contained “other” in foragers at oxidation resistance protein 1-like isoformX1). Thus, individual site class showed distinct age-related profiles. In some cases, pairs of sites within the same class showed similar phosphorylation patterns. However, even within the same pattern, different sites within the same class often showed variable pattern of phosphorylation.

### Prediction and identification of PKs in brains of nurse and forager bees

**Figure 4:**
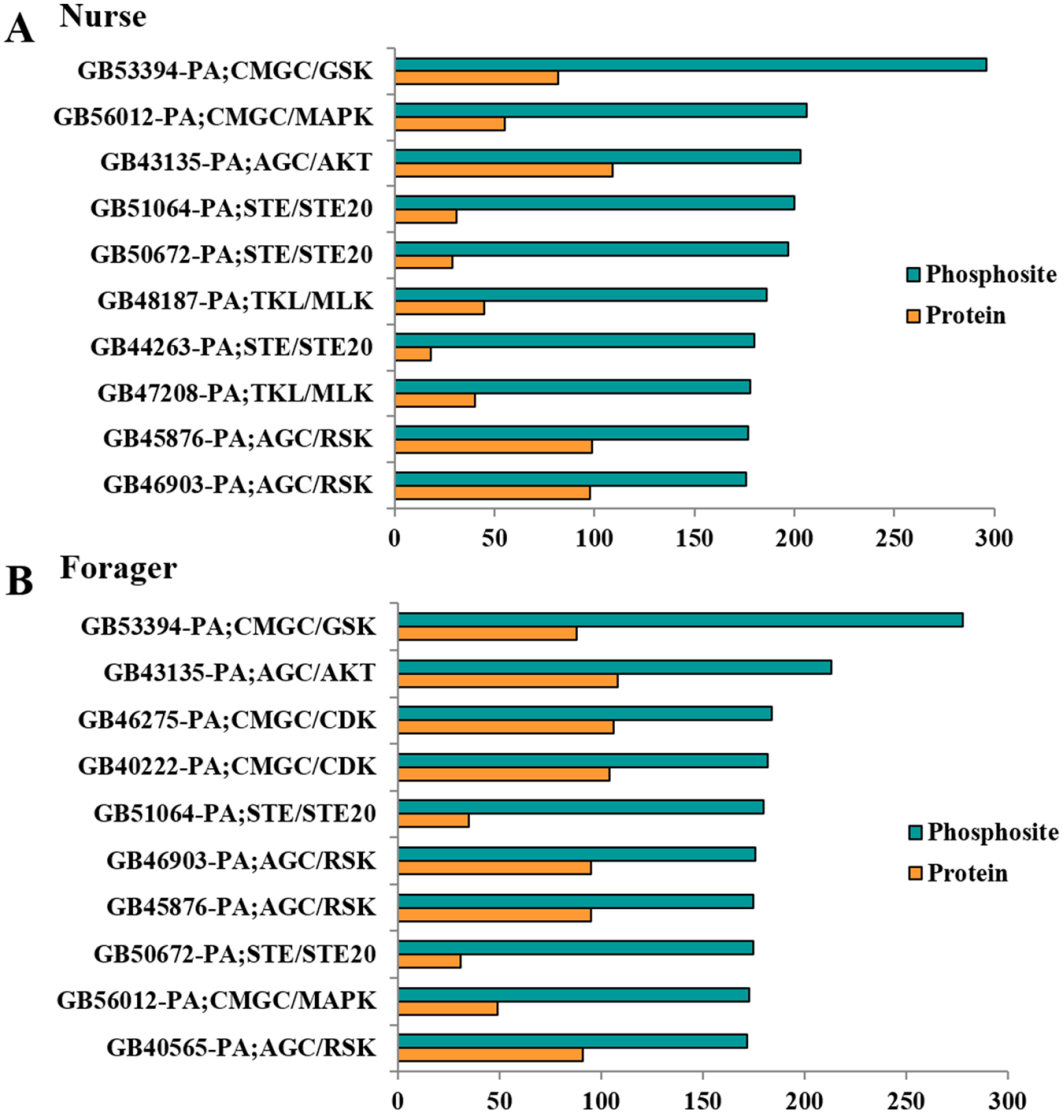
Group-based Prediction System (GPS) algorithm with the interaction filter, or *in vivo* GPS (iGPS). (**A**) The top 10 PKs with the most phosphosites by the prediction of iGPS in nurse bees. (**B**) The top 10 PKs with the most phosphosites by the prediction of iGPS in forager bees.

**Figure 5:**
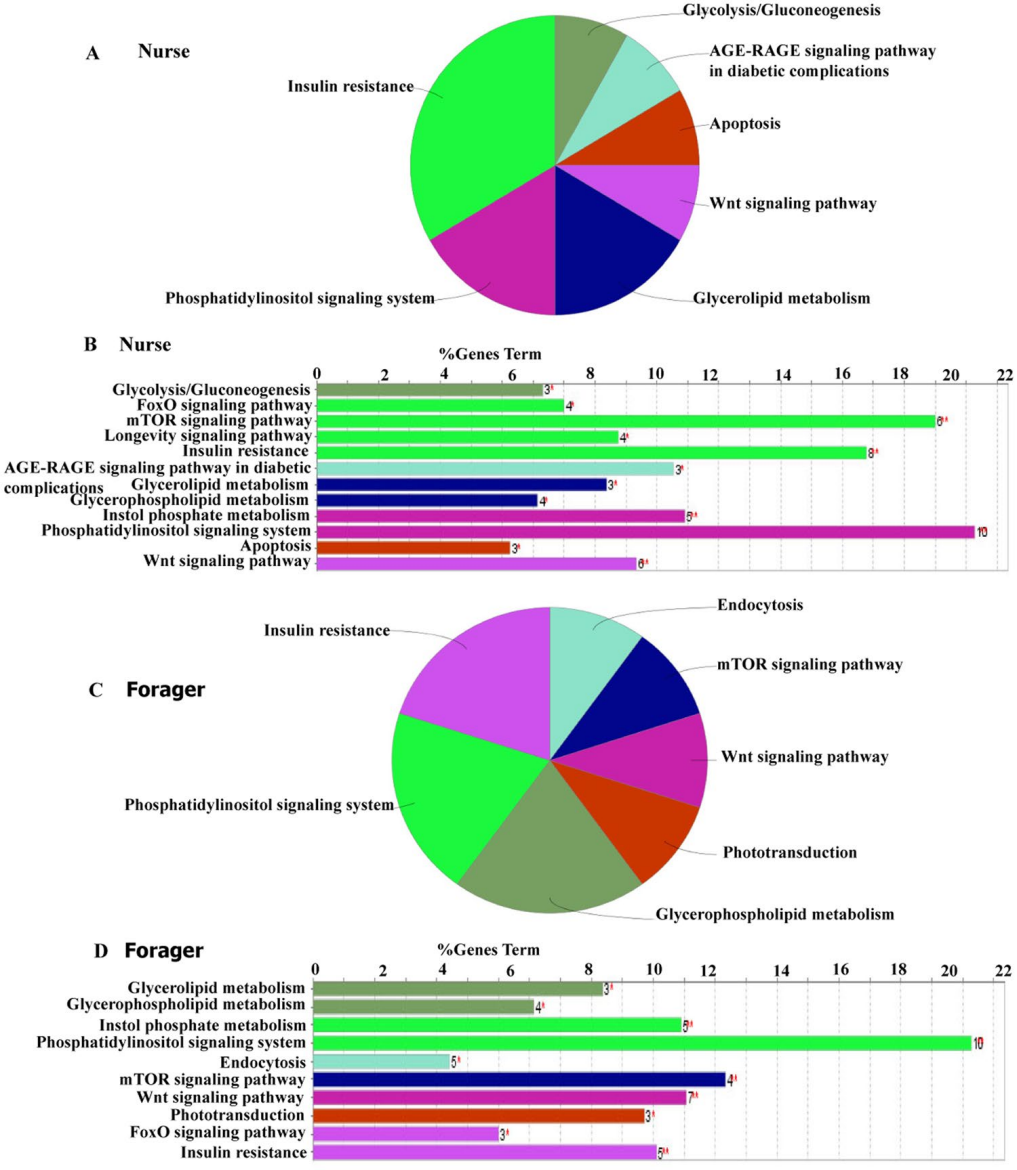
GlueGO analysis of protein kinases from the brain of honeybee workers (*A.m.ligustica*) which are identified in our experiment. GO biological process and KEGG pathway terms (first letter capitalized) specific for phosphoproteins from the brain of the honeybee workers (**p < 0.01;*p < 0.05). The numbers of corresponding genes associated with a specific term are indicated. The percentage of genes associated with a specific term is listed on the bars. (**A–D**) enriched ClueGO and KEGG pathway terms of phosphoproteins identified. A and B represents nurse bee and C and D represents forager bee.

Kinases are enzymes that modify proteins, changing the target protein’s activity in some way. They are the pivotal regulators of phosphorylation dynamics in cellular signaling^43^. Since PKs or kinome is not reported in the honeybee proteome, we predicted 231 PKs, divided into 10 groups and 103 families in the honeybee proteome (Supplemental Table 5). Based on the hypothesis that PKs in the same group or family would recognize similar motifs in the substrates for modification, the corresponding GPS 2.1 predictor was assigned for each honeybee PK if available. Finally, 179 PKs were selected with GPS predictors (Supplemental Table 6). For the phosphoproteomes of nurse and forager bees, we predicted 12,304 ssKSRs among the 140 PKs and 573 substrates for the 1,510 phosphosites with an average of 8.2 upstream PKs per phosphosites (Supplemental Table 7). For example, the nurse bees’ KSPN included 9,201 ssKSRs among the 140 PKs and 452 targets for 1,151 phosphosites with an average of 8 upstream PKs per phosphosites. From the networks, the top 10 PKs with the most phosphosites were selected and presented in Fig. 4A and B. Of all the 1,004 (nurse) and 958 (forager) phosphoproteins detected, 452 (45%) of those in nurse bees and 470 (49.1%) of those in forager bees were identified as potential substrates/kinase substrates for a particular PK or group/family of a PKs (Supplemental Table 8 and 9). Furthermore, we constructed the KSPNs of nurse and forager bees from the prediction results (Supplemental Figure 8A and B). In the constructed KSPN, 50 kinases were predicted as substrate and 99 as kinases in the nurse bees, and 51 kinases as substrate and 100 as kinase in the forager bees (Supplemental Table 9). In nurse bees, all PKs were significantly enriched in pathways associated with the foxo signaling pathway (*p* = 8.0e^−10^), mTOR signaling pathway (*p* = 2.0e^−10^) and wnt signaling pathway (*p* = 3.1e^−6^) (Supplemental Table 11). In forager bees, only phototransduction (*p* = 3.7e^−4^) was uniquely enriched, and all pathways enriched in nurse bees were also found in foragers (Supplemental Table 11). In addition, we experimentally identified 88 phosphoproteins as kinases, of which 60.2% (53 kinases) were commonly detected in both nurse and forager bees, whereas 20.5% (18 kinases) in nurse and 19.3% (17 kinases) in foragers were uniquely expressed (Supplemental Table 10). The pathways associated with Glycolysis/Gluconeogenesis (*p* = 4.4 e^−2^), AGE-RAGE signaling pathway in diabetic complications (*p* = 3.1e^−2^) and Apoptosis (*p* = 3.3e^−2^) were uniquely observed in nurse bees (Fig. 5A and B), whereas endocytosis (*p* = 3.4e^−2^), phototransduction (*p* = 3.0e^−2^), and mTOR signaling pathway (*p* = 3.7e^−3^) were unique to forager bees (Fig. 5C and D).

### Sequence and structure feature of phosphosites in nurse and forager brains

With regard to the position distribution of protein sequences, the phosphosites with predicted upstream PKs predominantly occurred in the C-terminal (Supplemental Figure 9A and E). In the secondary structures, the phosphosites with predicted upstream PKs were predominantly predicted to reside in amino acids with the coil than the α-Helix and β-Strand (Supplemental Figure 9B and F). As for the region preferences, the phosphosites with predicted upstream PKs preferred occurrence in the disorder region (Supplemental Figure 9C and G) and in the surface accessibility, they predominantly occurred in amino acids with the exposed domain (Supplemental Figure 9D and 9H).

Prediction of protein subcellular localization site is useful for screening candidate genes for their specific functions and for interpreting gene information^44^. Therefore, subcellular localization preferences of substrates for 9 different PKs groups were predicted in the nurse and forager bees. The phosphorylation events mainly occurred in the nucleus (Supplemental Figure 9I and J).

### Biological significance of phosphoproteins in nurse and forager brains

To explore the biological function of the identified phosphoproteins in the brains of nurse and forager bees, we analyzed and compared functional categories and biological pathways that were significantly enriched in both ages. In nurse bees, glycerolipid metabolic process (*p* = 3.2e^−4^), transport (*p* = 1.5e^−5^), vesicle-mediated transport (*p* = 4.1e^−3^), phosphorylation (*p* = 1.6e^−5^) and intracellular signal transduction (*p* = 1.3e^−6^) were significantly enriched (Supplemental Figure 10A and Supplemental Table 11). In the forager bees, glycerolipid metabolic process (*p* = 2.4e^−4^), phosphate-containing compound metabolic process (*p* = 3.0e^−5^), transport (*p* = 5.0e^−5^), vesicle-mediated transport (*p* = 1.1e^−3^), metal ion transport (*p* = 8.9e^−4^) and intracellular signal transduction (*p* = 7.6e^−4^) were significantly enriched (Supplemental Figure 10B and Supplemental Table 11). Moreover, phosphorylation process was only enriched in the nurse bees, whereas phosphate-containing compounds, metabolic processes, and metal ion transport were specifically enriched in forager bees. Mapping the identified phosphoproteins into biological pathways could better the understanding of the phosphorylation dynamic in the pathways. For the pathways of inositol phosphate metabolism (Supplemental Figure 11), although much of the network was commonly utilized in the central signaling pathway, age-specific patterns were also apparent^45, 46^. For example, 1-phosphatidyl-1D-myo-inositol5P, 1D-myo-inositol-1,3,4P3 and myo-Inositol were commonly utilized in both ages whereas D-Glucose-6P and dihydroxyacetone phosphate was phosphorylated only in the nurse brain and absent in forager bees.

**Figure 6:**
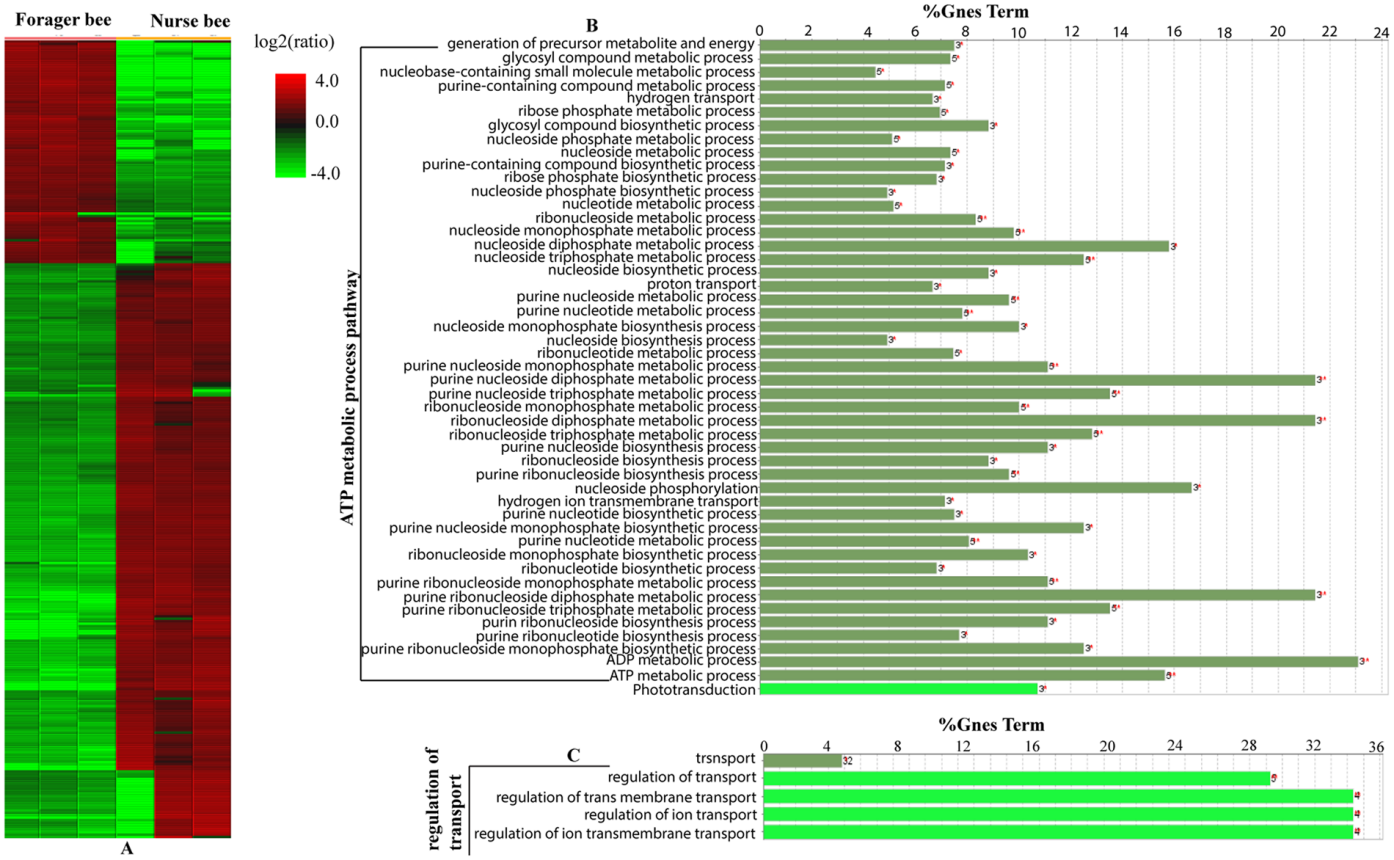
Quantitative proteome comparison during the development of bee brain (fold change ≥2 and p < 0. 05). (**A**) Hierarchical clustering of the differentially expressed proteins (fold change >2 and P < 0.05); the columns represent the replicates in each of the nurse and forager bees, and the rows represent the individual proteins. The up- and down-regulated proteins are distinguished by red and green color, respectively. The color intensity changes with the protein expressional level as indicated on the bar. (**B** and **C**), Enriched functional groups and pathways of up-regulated proteins in the nurse and foraging bee brain respectively.

To evaluate the phosphoproteome profile change between the brains of nurse and forager bees, 327 phosphoproteins (26.6% of all identified 1,244 phosphoproteins) were found differentially expressed. Of those differential proteins, 101 (30.1%) and 226 (69.1%) were up-regulated in nurse and forager bees, respectively (Fig. 6A and Supplemental Table 12). The up-regulated phosphoproteins in nurses were significantly enriched in the functional categories related to transport (*p* = 1.6e^−2^) and regulation of transport (*p* = 2.3e^−3^) (Fig. 6B and Supplemental Table 11). Whereas ATP metabolic process (*p* = 2.2e^−2^) and phototransduction (*p* = 2.5e^−2^) were significantly enriched in forager bees (Fig. 6C and Supplemental Table 11).

### Verification of differentially expressed proteins at the level of mRNA and protein

**Figure 7:**
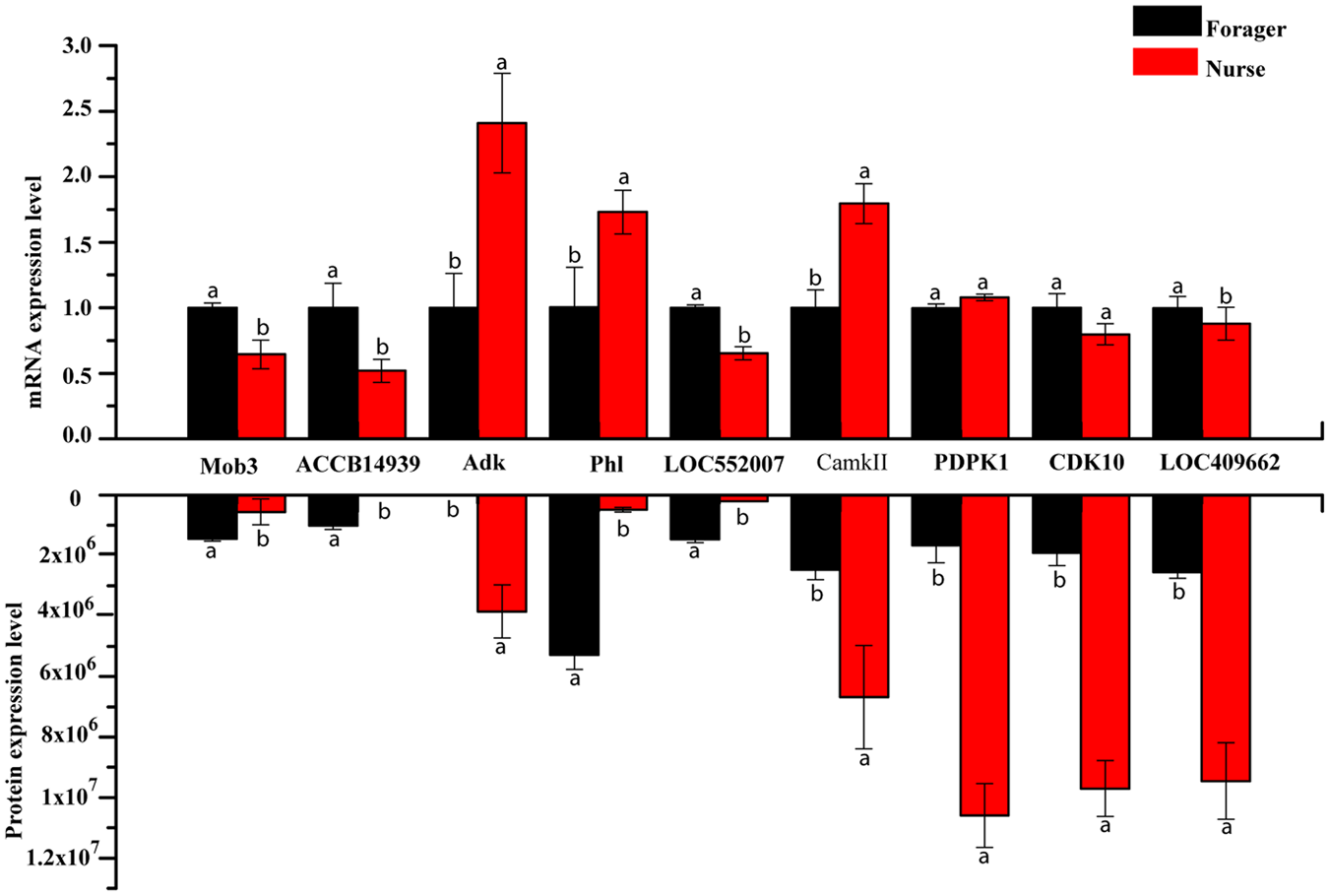
Verification of differentially expressed phosphoproteins at mRNA level. Nine differential proteins (*Mob3, ACCB14939, Adk, Phl, LOC552007, CamkII, PDPK1, CDK10* and *LOC409276*) were selected for qRT-PCR analysis. The color bars represent the relative expression values of proteins in nurses and foragers brains. Different letters indicate significant difference at (p < 0.05), and the error bar indicated standard deviation. Abbreviated proteins and specific primers used for qRT-PCR were provided in Supplemental Table 1.

To test the differentially expressed proteins between the brain of nurse and forager bees at gene level, 9 differential proteins related to ATP metabolic process, phototransduction, ribonucleoside triphosphate metabolic process, glycerophospholipid metabolic process, Wnt signaling pathway, phosphorylation, Inositol phosphate metabolism, phosphate-containing compound metabolic process, were selected for qRT-PCR analysis. Of the 9 proteins in both ages *Mob3*, *ACCB14939*, *Adk*, *Phl*, *LOC552007*, *CamkII*, *PDPK1*, *CDK10* and *LOC409276* were significantly different between the two ages and in line with their protein expression tendencies (Fig. 7).

## Discussion

Temporal age-related division of labor in nurse and forager honeybees is an essential social behavior that supports the well-organized social order^47^. To achieve the age-dependent division of labor, the nurse and forager bees require an efficient nervous system to coordinate the complex social and behavioral interactions within the colony^9^. To better understand how phosphorylation networks regulate this behavior transition, the phosphoproteome in the brains of nurse and forager honeybees were characterized. A hitherto unknown depth of phosphoproteome and kinome were defined in the honeybee brain at two different physiological states. The higher number of phosphopeptides, phosphosites, phosphoproteins and PKs identified in the brains of nurse bees indicate that the nurse bees may need deeply committed phosphoproteome in building molecular and neural structures. This is because reversible phosphorylation of Ser, Thr and Tyr residues is a prominent signaling mechanism to enable spatial and temporal regulation of the activation states, conformations or binding interactions of proteins, and thereby regulates diverse downstream effects^42, 48^. The high occupancy of sites preferentially phosphorylated on disorder regions is likely to activate upstream kinase activity favoring disorder regions^49^. The phosphopeptides predominantly modified sites in the vicinity of their C-terminal, and occurred in amino acids with the exposed portion^34^ suggesting that phosphorylation tends to occur independent of structure in unstimulated cells and that proteins fold into unique structures based upon primary amino acid sequences like in other organisms^50^. Thus, protein phosphorylation events regulate a wide scenario of key cellular signaling pathways and functional classes in the honeybee brain to fit with the age-dependent physiological roles.

Many cellular activities are controlled by multiple phosphosites on proteins that show different regulatory trends^34, 42, 51^. It is reflected in our data that some proteins with a high number of phosphosites differ in the extent of residue phosphorylation in both nurse and forager bees. Examples of such proteins were neurofilament heavy polypeptide, which is implicated in letting nerve cells to establish and maintain a remarkably complex set of highly asymmetrical cellular extensions^52^, and neurofilament phosphorylation may regulate the interaction of neurofilament with other neuronal structures^53^. In microtubule-associated protein futsch and neurofilament heavy polypeptide proteins the Ser and Thr residues are phosphorylated and dephosphorylated interchangeably in different sites in the nurse and forager honeybees (Fig. 2). These phosphorylation sites might represent phosphoproteins that reflect their phosphorylation/dephosphorylation cycles of the brain development and likely to prime the protein functions in tuning neural activity in the different ages of honeybees.

PKs are key regulators in protein phosphorylation, which modify their target proteins by chemically added phosphate groups to specific amino acids on the Ser, Thr and Tyr residues, and the function of PKs is decided by the sequence motif on the substrate or site class^31^. The age-specific presence of site classes (Acidic, Proline-direct, Tyrosine and “Other”) and the distinctive age-specific kinome profiles between the nurse and forager bees indicate that signaling in the neural activity depends upon a wide repertoire of up and downstream cellular signaling regulatory processes. The distinct kinase classes between the nurse and forager bees, especially the tyrosine class only found in nurse bees, suggest that different kinase cascades occur in the honeybee brain according to age. It is in line with the finding hat tyrosine phosphorylation plays a significant role in honeybee brain development as in mammals^54^, and is vital for the transition from nursing to foraging, which demands brain cell differentiation and development^19, 22^. Notably, the activity of some proteins is likely regulated by multiple kinases representing discrete signaling networks in the two ages of bees. For instance, 3 site classes at 6 phosphosites in spectrin beta chain isoform were observed in the two ages of honeybee, 3 site classes in nurse bee and 2 site classes in forager bee were covered 8 phosphosites in oxidation resistance protein 1-like isoform. Comparing with only two kinase classes previously reported in the hypopharyngeal glands of honeybees^22^, the four kinase classes found here in the central nervous system are indicative of the fact that more complicated and advanced signaling mechanisms are likely developed in the highly advanced center of the brain^22^. This is further manifested in the fact that tissue-unique phosphorylation events have evolved to underline their distinct physiology and the knowledge that kinase expression is not conserved across the honeybee organ or tissues, which is in line with known mechanism in the *Drosophila* ^55^. In forager bees, the high abundance levels of proteins related to cAMP signaling, such as cAMP-dependent protein kinase (PKA), suggest that phosphorylation is essential in regulating olfactory associative learning and memory, as in *Drosophila* ^55, 56^. Moreover, the uniquely expressed PKs in nurse bees related to phosphofructokinase suggest its role in the regulation of glycolysis in different components of the brain, similar to its function/role in rats^57^. Although the honeybees are claimed as a model insect, its kinome is still unknown, thus hindering the downstream activity analysis of phosphorylation. Here, the 179 PKs with GPS predictors used to predict their site-specific kinase–substrate relations by GPS algorithm (231 PKs in the whole honeybee proteome) are the first reports of the honeybee kinome and are vitally important in neurological signaling. The different phosphosites detected in nurse and forager bees as target substrates or kinases suggests that distinct cerebral activity has evolved depending on the physiological state of the bees. Specifically, of the top 10 PKs with the most phosphosites in the predicted PKs, CMGC (CMGC/DYRK, CMGC/GSK, and CMGC/CDK) with highest number of phosphosites in both ages of bees suggest their vital roles implicated in cell cycle/cell division (e.g. CMGC/CDK) and signal transduction (e.g. CMGC/GSK)^39^. All these observations are helpful to gain novel insights into the signaling network that functions in bee brain development and functionality.

Investigating phosphorylation signaling implicated in biological pathways and functional classes is necessary in understanding the biological activities of signaling transduction in an organism^31^. Here, a wide spectrum of pathways and functional classes was enriched by the phosphoproteins, manifesting the fact that the age-related signaling architectures have been evolved in the worker bees’ brain to drive their cerebral functionality. For instance, the wnt signaling pathway involved in both honeybee ages is a group of signal transduction pathways which drives the flow of signals from outside to inside the cell via cell surface receptors, which thus reinforces the neural functionality in brain cells^58^. This pathway is highly involved in transcription factor AP-1, calcium/calmodulin-dependent PK II and axin-1 proteins, suggesting their critical roles in signal transduction to support brain interaction with environment signals such as queen substance, chemicals, colors, and temperature alterations^59^. The uniquely enriched pathways and functional classes, such as phosphorylation, AGE/RAGE and glycolysis/gluconeogenesis in nurse bees suggest their importance for the brain’s cellular maturation and the development of cerebral structure to support the age-specific tasks of nurse bees^60^. It is reported that the high rate of protein synthesis in the brain of nurse bees is a key defining characteristic of this age to differentiate and develop the brain, as the transition from nursing to foraging involves changes in brain structure^23^. To this effect, the highly expressed proteins involved in pathways and functional classes such as phosphorylation, transport and regulation of transport are indicative of the fact that phosphorylation plays key roles for transporting those proteins necessary for the changes in brain structure^22^. The uniquely expressed AGE/RAGE signaling pathway in nurse bees suggests the activation of multiple intracellular pathways involving in NADPH oxidase, PKC, and MAPKs, and then resulting in NF-kappaB^61, 62^. Moreover, the uniquely expressed glycolysis/gluconeogenesis pathway in nurse bees implies its central role in producing important precursor metabolites and synthesizing glucose (this glucose is required by the brain for its proper functioning) from non-carbohydrate precursors necessary^63^. In foragers, the brain is well-developed^18^ and the strongly and uniquely expressed functional classes and pathways related to metal ion transport, ATP metabolic process and phototransduction suggest that they are vital in responding to sequential environmental signals and information, as neurotransmitters to sufficiently support guarding and foraging activities^64, 65^. Furthermore, the uniquely enriched functional class associated with metal ion transport in forager bees indicates its importance in transporting ion metals to the brain, as some metals are particularly important for brain function^66^. As is well-known, all metabolic processes are life-sustaining vital chemical processes that sustain energy production and cell growth^64, 67^. In foragers, the highly expressed phosphoproteins related to ATP-metabolic processes are assumed to produce highly energetic molecular ATP^64^ that powers most cellular reactions for neural activity, which is important for the bees in order to travel long distances for foraging activity. The unique phototransduction pathway by the phosphoproteins in the forager brain suggests that phosphorylation is vital for visual perception and information acquisition of flower colors and patterns and the route to food sources during field foraging activities^65^. This is consistent with the fact that calcium/calmodulin-dependent protein kinase II and protein kinase C is involved in the phototransduction pathway via the intracellular Ca^+2^ signal transduction in the mushroom body of the worker bee brain ^68^ and important to shut off the light response, as found in/known for *Drosophila* ^69^. The multiplicity of the enriched functional groups and pathways by the phosphoproteins in the two ages suggest that phosphorylation signaling regulates a wide cascade of the biological roles in the central nervous system of the honeybee brain to sustain age-dependent roles as nurse or forager bees.

Mapping the identified proteins into a canonical pathway can gain deep insight into the biological significance that a specific protein played at the pathway-centric level^11^. For instance, the phosphorylated glycerophospholipid metabolism process in the nurse and forager brains indicates its role in promoting glycerophospholipid synthesis to ensure its function as a reservoir for second messengers in the neural membrane and its involvement in modulating transport activity^70^. This pathway is highly controlled by phosphorylation reflected as age-specific expressions of different glycerophospholipids subunits. A higher number of subunits of inositol phosphate, such as D-Glucose-6P and dihydroxycetone phosphate, were phosphorylated in the brain of nurse bees, as compared to the number in forager bees. This observation indicates that different protein species are phosphorylated to relay and amplify the signal in the inositol phosphate metabolism pathways in the nurse and forager bees to drive the different cerebral functions^22, 71^. The validated expression tendency between the phosphoproteins and their encoding genes *Mob3*, *ACCB14939*, *Adk*, *Phl*, *LOC552007*, *CamkII*, *ARGK*, *PDPK1* and *LOC409276* suggests that protein phosphorylation and gene expression may have parallel directions in regulating functionality in the brain, thus providing sound clues to investigating the functions of modified specific proteins in regulating the physiological changes of the brain.

## Conclusion

This work represents the first and most in-depth coverage of the *in vivo* phosphoproteome in the honeybee brain and documented 4,138 phosphosites from 1,244 phosphoproteins. The dynamic alteration of phosphosites and site abundance levels of the phosphoproteins in the brains of nurse and forager bees indicates that the age-dependent labor division of the honeybee requires specialized phosphorylation networks to consolidate their unique neural biology. This age-dependent phosphoproteomic further reflects that the unique biological pathways and kinase activities are employed for the neurobiological activities in the brain to validate with the biological duties as nursing and foraging bees. Furthermore, the identification of PKs and kinase-specific substrates is vital for understanding the regulatory mechanisms of protein phosphorylation, especially in regulating the neural activity to prime the age-related labor division in honeybee workers. Hence, our results gain novel insights into the range of functions regulated by phosphorylation at different time points in the honeybee brain. This data provides a trustworthy basis for future studies of the functions of these signal transduction pathways in honeybee neurobiology, as well as in neurobiology of other social insects.

## Electronic supplementary material

Supplementary information

Table S1

Table S2

Table S3

Table S4

Table S5

Table S6

Table S7

Table S8

Table S9

Table S10

Table S11

Table S12
