# Supplementary material for: Phosphoproteome Analysis Reveals Phosphorylation Underpinnings in the Brains of Nurse and Forager Honeybees (Apis mellifera)
Source: Sci Rep. 2017 May 16;7:1973. doi: 10.1038/s41598-017-02192-3 (PMC5434016; doi:10.1038/s41598-017-02192-3)
Supplement: Supplementary file 1 — Supplementary information [file 41598_2017_2192_MOESM1_ESM.pdf]

Phosphoproteome Analysis Reveals Phosphorylation Underpinnings in the Brains of Nurse and Forager Honeybees (*Apis mellifera*)

Gebreamlak Bezabih[1, #]:gbtesfay@yahoo.com

Han Cheng[2, #]: chenghan@zzu.edu.cn

Bin Han[1]: hanbin_bee@163.com

Mao Feng[1]: fengm622@163.com

Yu Xue[3]: xueyu@hust.edu.cn

Han Hu[1]: wuhan_hh@126.com

Jianke Li[1, *]: apislijk@126.com

SUPPLEMENTARY FIGURES (Figs S1-S11)

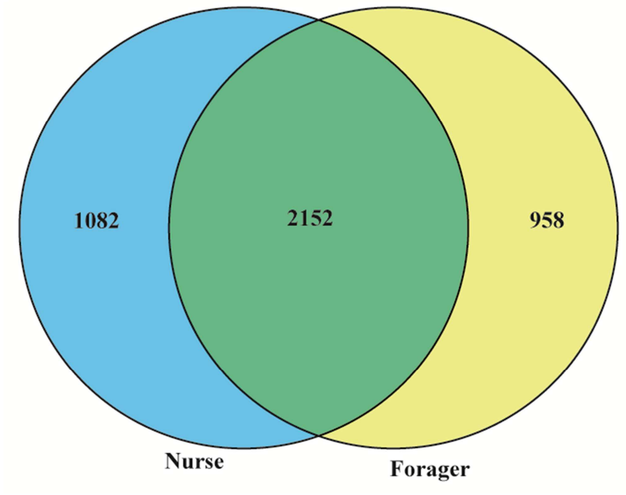

Supplemental Figure 1. Summary of phosphopeptides found in nurse and forager honeybee brain phosphoproteome. 1,082 unique to nurse, 958 unique to forager and 2,152 shared between the nurse and forager honeybees.

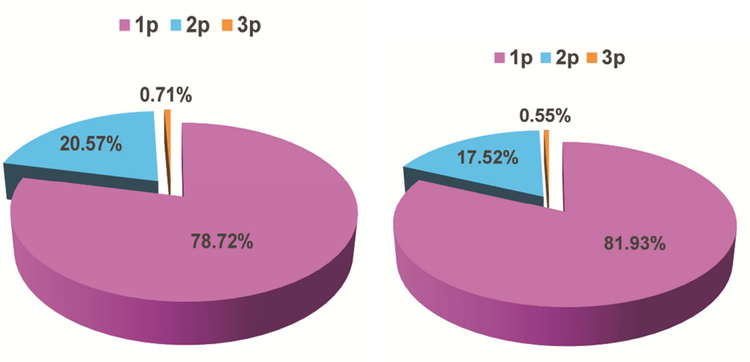

A B

Supplemental Figure 2. The distribution of phosphopeptides depending on the number of phosphosites identified in the honeybee brain. (A) Stands for nurse bee and (B) for forager bees. The 1p, 2p and 3p represent a phosphopeptide with one, two and three phosphosites, respectively.

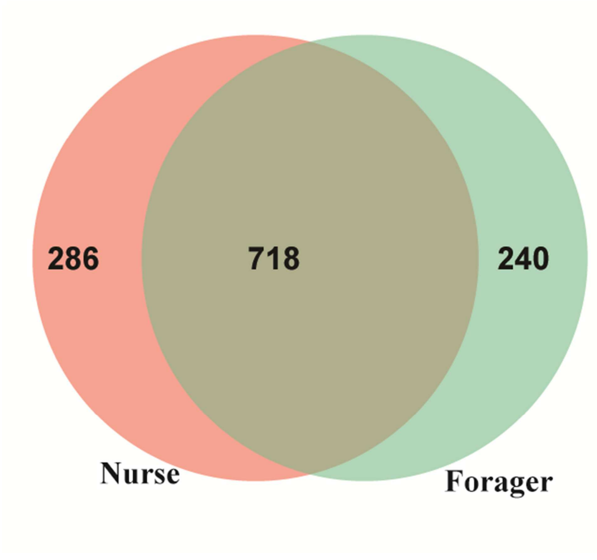

Supplemental Figure 3. Summary of the phosphoproteins found in nurse and forager bees brain phosphoproteome. The 286 were unique to nurse, 240 unique to forager and 718 shared between the nurse and forager honeybees.

WKDEDDsDDEGGMEGSSHDLEEMMR

gi|571575529

Q: N/F = 2.83/1

Note: - Q= quantification and N/F =Nurse vs. Forager

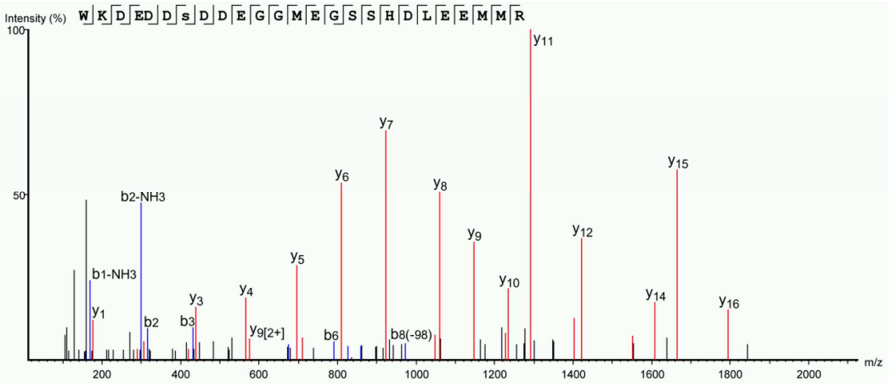

Nurse: mass per charge (m/z)=1490.5312, z=2, Retantion time (RT)=101.23, Peptide Score=63.44, Ascore=1,000.00, Probability: 100%

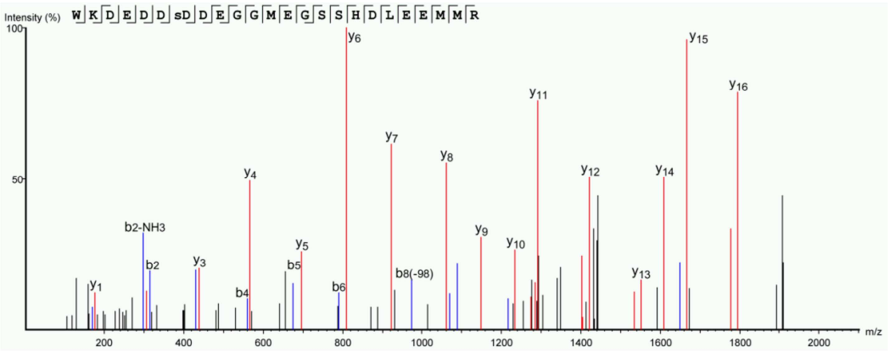

Forager: mass per charge (m/z)=1490.5286, z=2, Retantion time (RT)=101.94, Peptide Score=67.98, Ascore=1,000.00, Probability: 100%

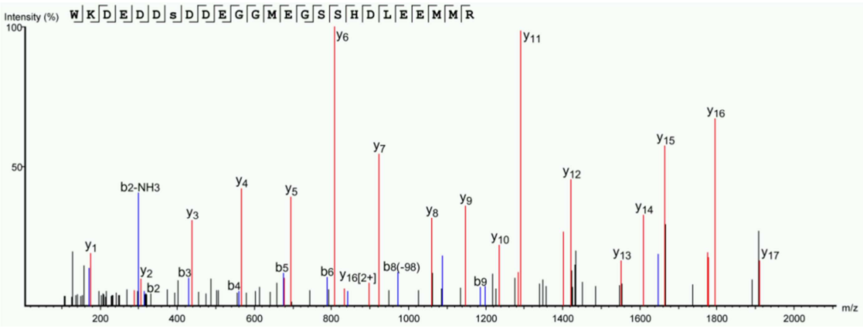

Validated: mass per charge (m/z)=1490.5171, z=2, Retantion time (RT)=101.05, Peptide Score=83.28, Ascore=1,000.00, Probability: 100%

Source of the document: Peaks software and Scaffold PTM

SLsPSASLATLR

gi|571538775

Q: N/F = 1.8/1

Note: - Q= quantification and N/F =Nurse vs. Forager

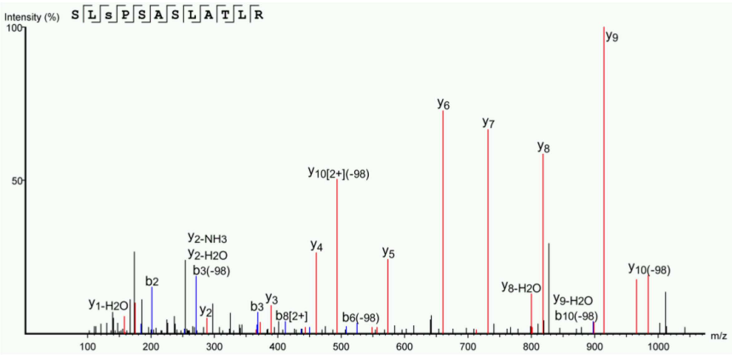

Nurse: mass per charge (m/z)=641.8224, z=2, Retantion time (RT)=78.56, Peptide Score=49.56, Ascore=1,000.00, Probability: 100%

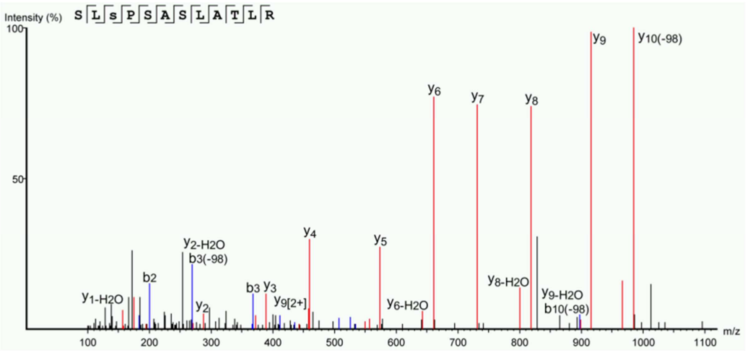

Forager: mass per charge (m/z)=641.8217, z=2, Retantion time (RT)=78.68, Peptide Score=49.11, Ascore=1,000.00, Probability: 100%

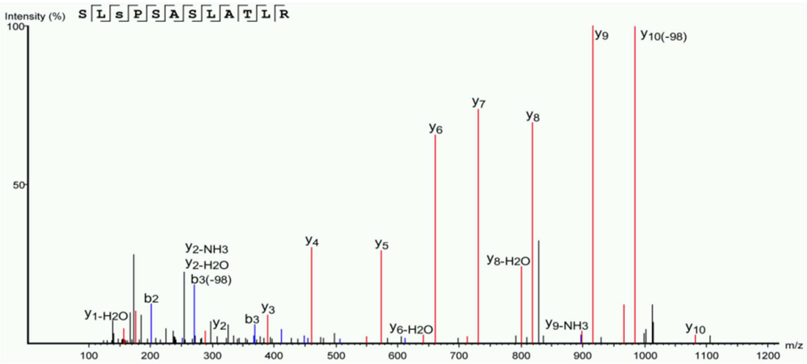

Validated: mass per charge (m/z)=641.8201, z=2, Retantion time (RT)=79.35, Peptide Score=40.48, Ascore=1,000.00, Probability: 100%

Source of the document: Peaks software and Scaffold PTM

NALQGFsPNHKITSFAEAK

gi|571522727

Q: N/F=0.06/1

Note: - Q= quantification and N/F =Nurse vs. Forager

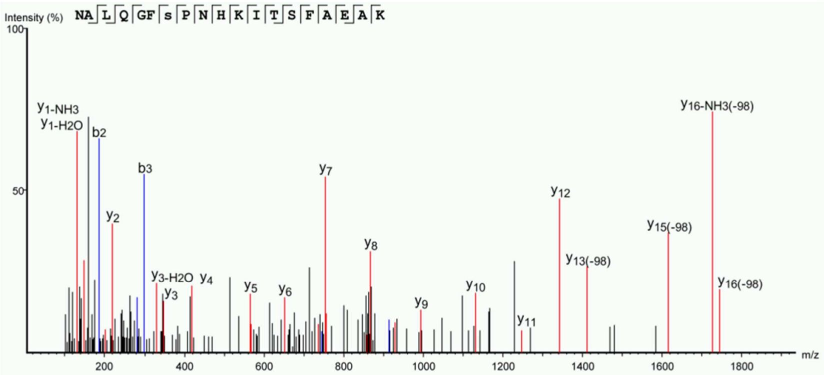

Nurse: mass per charge (m/z)=714.0081, z=3, Retantion time (RT)=79.99, Peptide Score=34.46, Ascore=1,000.00, Probability: 100%

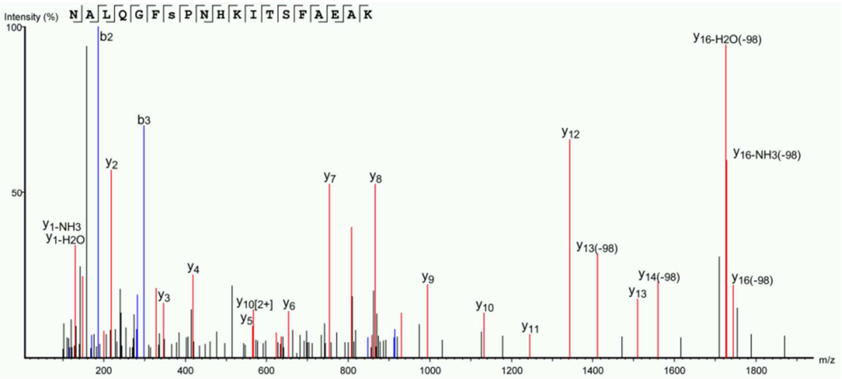

Forager: mass per charge (m/z)=714.0099, z=3, Retantion time) RT=79.60, Peptide Score=51.85, Ascore=1,000.00, Probability: 100%

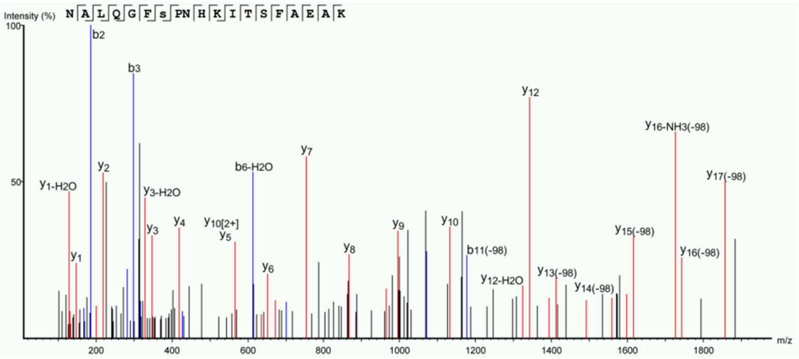

Validated: mass per charge (m/z)=714.0079, z=2, Retantion time (RT)=79.26, Peptide Score=48.24, Ascore=1,000.00, Probability: 100%

Source of the document: Peaks software and Scaffold PTM

AREEEDLLEHEsPENKYK

gi|571525698

Q: N/F=0.08/1

Note: - Q= quantification and N/F =Nurse vs. Forager

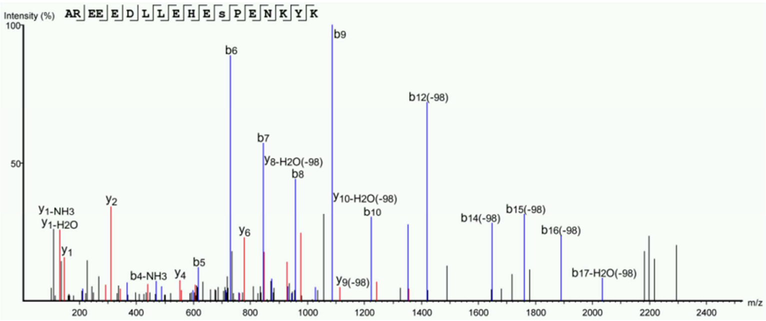

Nurse: mass per charge (m/z)=766.0059, z=3, Retantion time (RT)=37.74, Peptide Score=34.46, Ascore=1,000.00, Probability: 100%

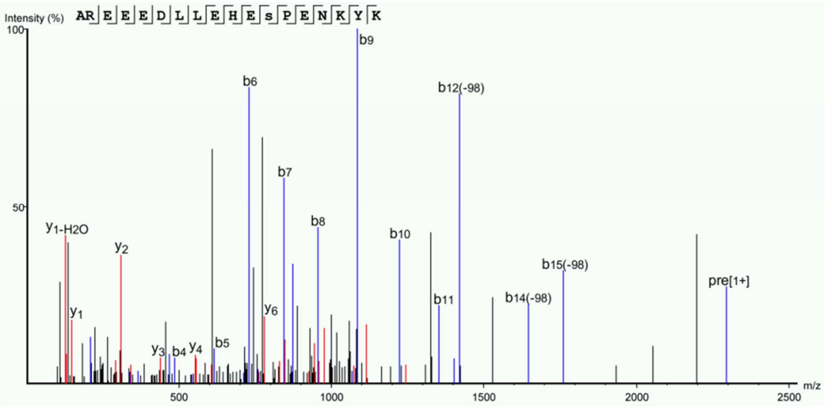

Forager: mass per charge (m/z)=766.0079, z=3, Retantion time (RT)=35.84, Peptide Score=50.11, Ascore=1,000.00, Probability: 100%

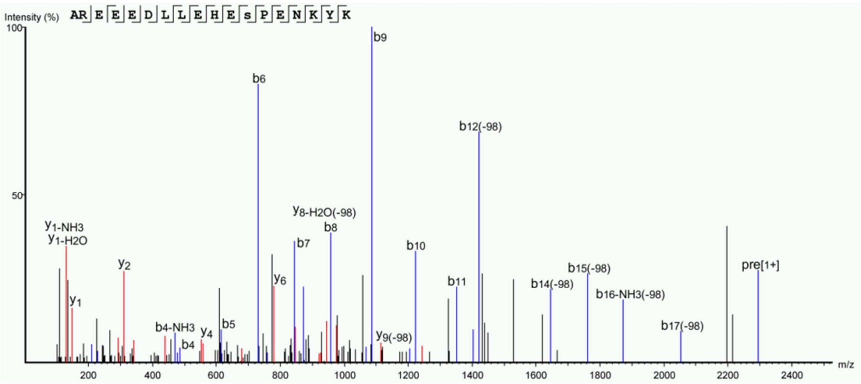

Validated: mass per charge (m/z)=766.0024, z=3, Retantion time (RT)=36.39, Peptide Score=49.11, Ascore=1,000.00, Probability: 100%

Source of the document: Peaks software and Scaffold PTM

KPPtPQQTVPISETTALTEK

gi|571574999

Q: N/F=0.34/1

Note: - Q= quantification and N/F =Nurse vs. Forager

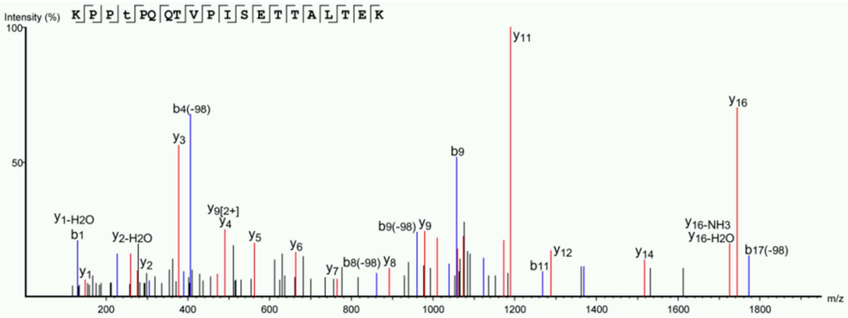

Nurse: mass per charge (m/z)=1123.5603, z=2, Retantion time (RT)=64.40, Peptide Score=42.54, Ascore=1,000.00, Probability: 100%

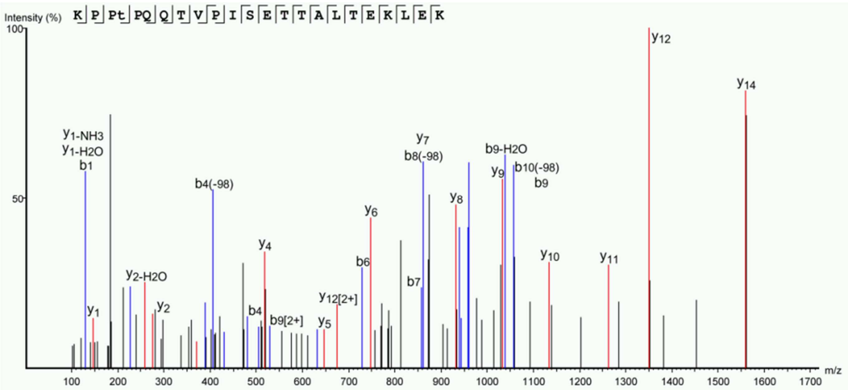

Forager: mass per charge (m/z)=1123.5615, z=2, Retantion time (RT)=65.25, Peptide Score=56.85, Ascore=1,000.00, Probability: 100%

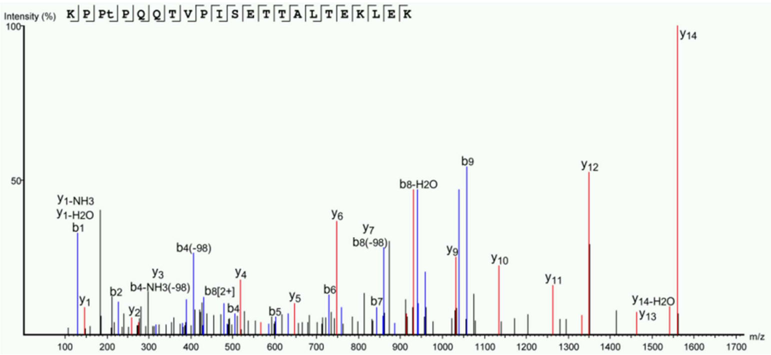

Validated: mass per charge (m/z)=1123.5610, z=3, Retantion time (RT)=66.38, Peptide Score=60.12, Ascore=1,000.00, Probability: 100%

Source of the document: Peaks software and Scaffold PTM

SHPSNMAGtPPHK

gi|328787452

Q: N/F=8.12/1

Note: - Q= quantification and N/F =Nurse vs. Forager

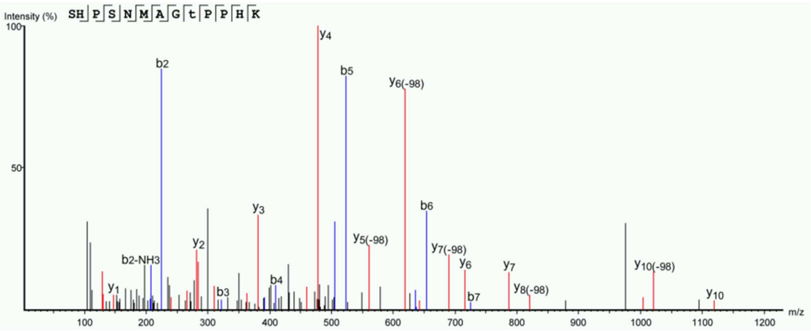

Nurse: mass per charge (m/z)=480.8716, z=3, Retantion time (RT)=10.90, Peptide Score=39.47, Ascore=1,000.00, Probability: 100%

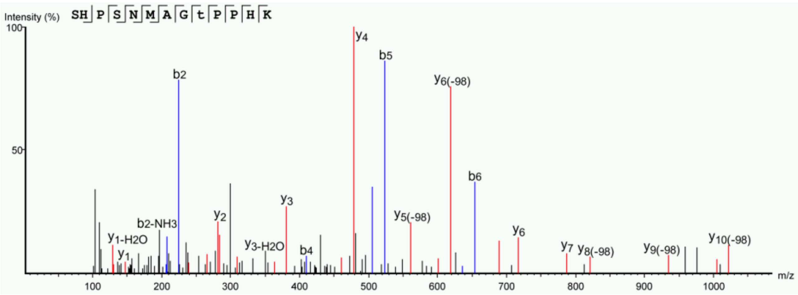

Forager: mass per charge (m/z)=480.8731, z=3, Retantion time (RT)=11.05, Peptide Score=45.39, Ascore=1,000.00, Probability: 100%

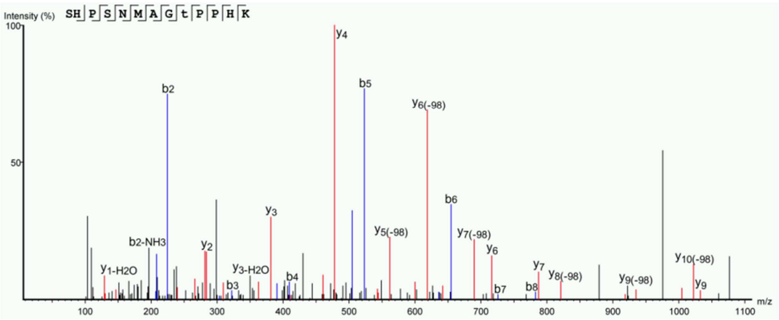

Validated: mass per charge (m/z)=480.8719, z=3, Retantion time RT=10.78, Peptide Score=49.12, Ascore=1,000.00, Probability: 100%

Source of the document: Peaks software and Scaffold PTM

TAGTTFMMTP**y****VVTR**

gi|571516301

Q: N/F=4.04/1

Note: - Q= quantification and N/F =Nurse vs. Forager

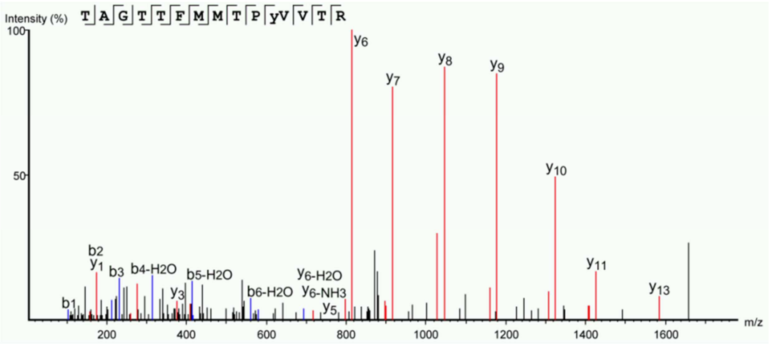

Nurse: mass per charge (m/z)=878.3922, z=3, Retantion time (RT)=102.38, Peptide Score=38.11, Ascore=1,000.00, Probability: 100%

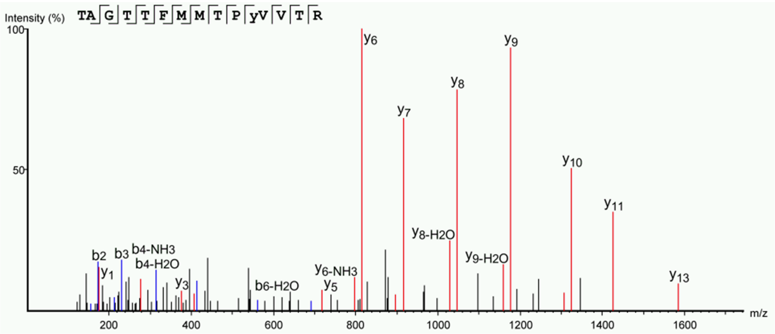

Forager: mass per charge (m/z)=878.3907, z=2, Retantion time (RT)=104.01, Peptide Score=37.39, Ascore=1,000.00, Probability: 100%

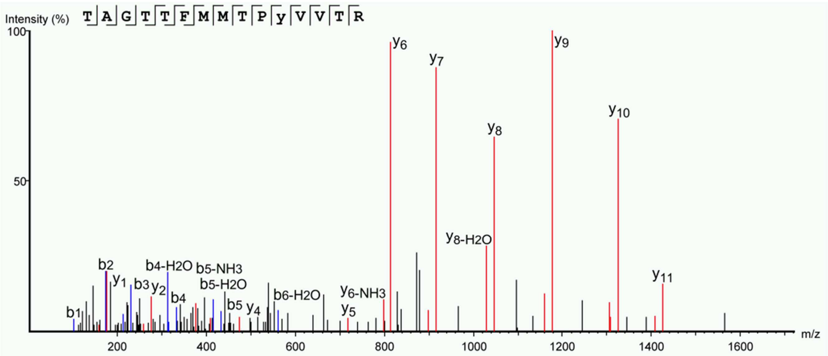

Validated: mass per charge (m/z)=480.8725, z=3, Retantion time (RT)=102.78, Peptide Score=53.45, Ascore=1,000.00, Probability: 100%

Source of the document: Peaks software and Scaffold PTM

HLVKGEPNVSyICSR

gi|571550120

Q: N/F=0.38/1

Note: - Q= quantification and N/F =Nurse vs. Forager

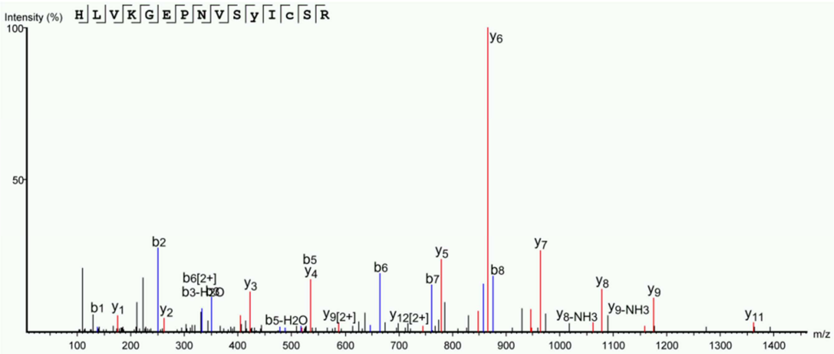

Nurse: mass per charge (m/z)=613.6214, z=3, Retantion time (RT)=38.35, Peptide Score=47.66, Ascore=1,000.00, Probability: 100%

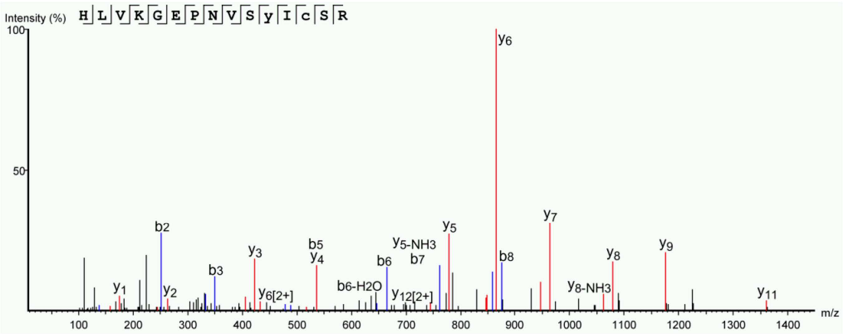

Forager: mass per charge (m/z)=613.6237, z=3, Retantion time (RT)=38.72, Peptide Score=61.71, Ascore=1,000.00, Probability: 100%

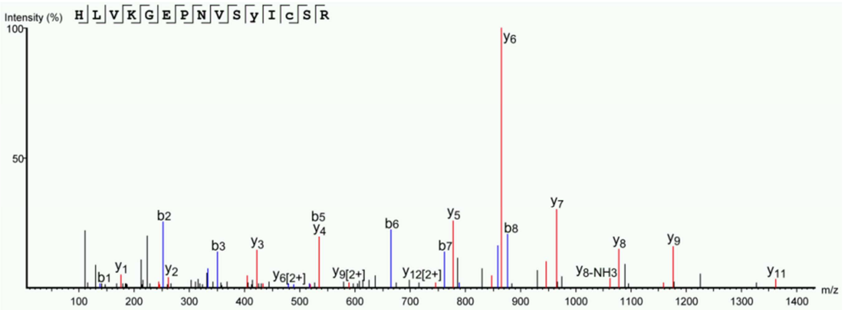

Validated: mass per charge (m/z) =613.6225, z=3, Retantion time (RT) =38.78, Peptide Score=60.19, Ascore=1,000.00, Probability: 100%

Source of the document: Peaks software and Scaffold PTM

Supplementary Figure 4. Mass spectra that validated the phosphorylation peptides in nurse and forager brain of the honeybee worker (*A. m. ligustica*) by comparison of mass spectra of artificially synthsis of corresponding phosphorylation peptides.

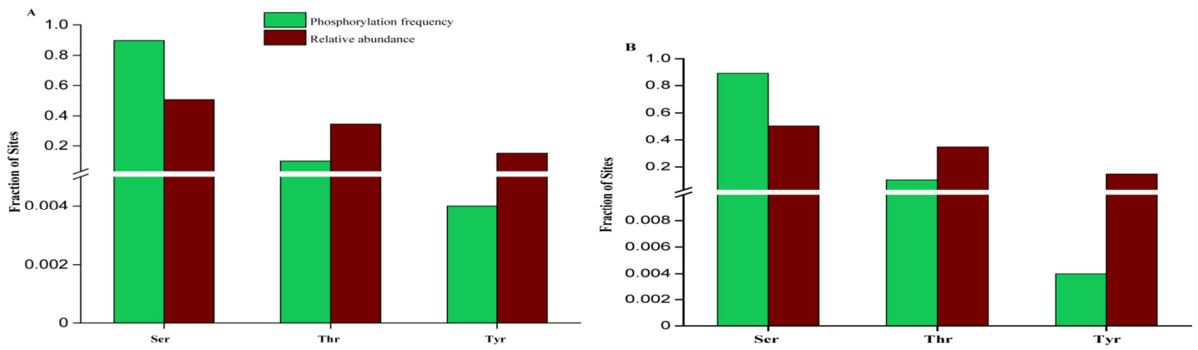

Supplemental Figure 5. Distribution of age-dependent phosphosites in the brain of honeybee workers (*A. m. ligustica*) at two ages related to phosphoproteins and their relative likelihood of phosphorylation. A. The relative frequencies of Ser, Thr, and Tyr within all phosphoproteins and their relative likelihood of phosphorylation in the forager honeybee. B. The relative frequencies of Ser, Thr, and Tyr within all phosphoproteins and their relative likelihood of phosphorylation in the nurse honeybee.

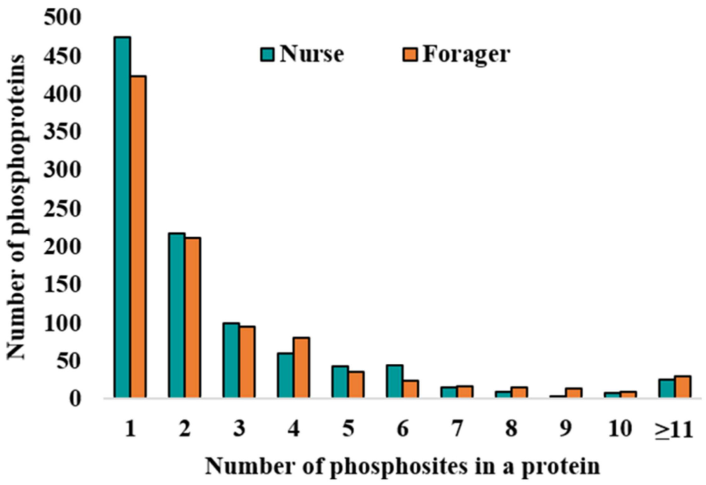

Supplemental Figure 6. The distribution of phosphoproteins based on the number of phosphosites identified in the nurse and forager honeybees

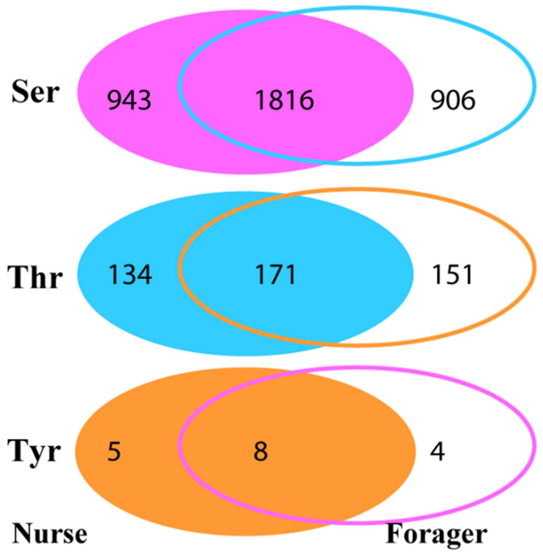

Supplemental Figure 7. Comparison of the phosphosites in different residues (Ser,Thr and Tyr) in the nurse bees and forager bees.

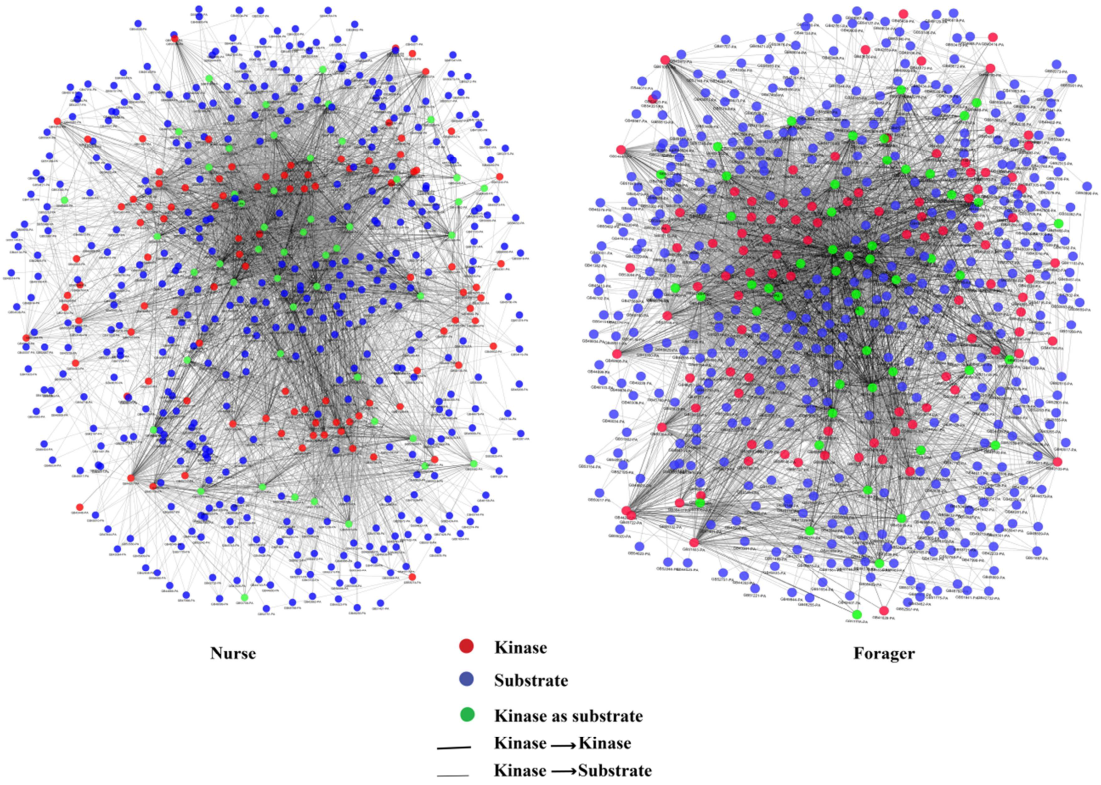

Supplemental Figure 8. The constructed kinase-substrate phosphorylation network in the nurse and forager honey bees based on the identified phosphopeptides according to the method described in construction of kinase and substrate interaction network

.

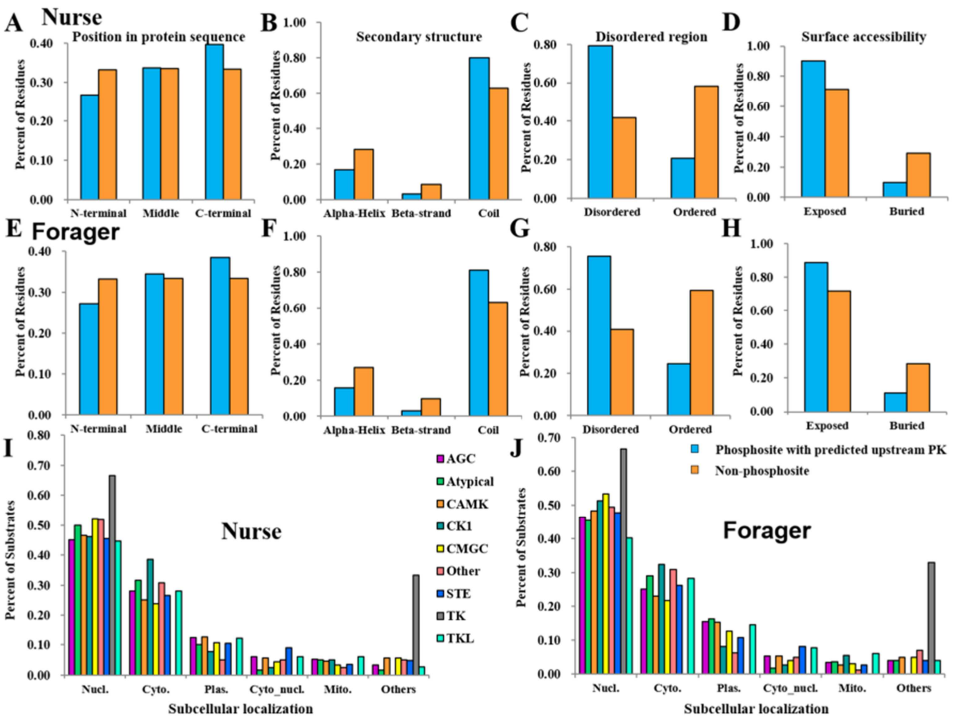

Supplemental Figure 9. The sequential and structural preferences of two types of sites including predicted phosphosites and non-phosphosites in several aspects. A and E denote position in protein sequence. B and F represent secondary structure states. C and G indicate disordered region. D and H stand for surface accessibility for nurse bees and forager bees. The subcellular localization preferences of substrates for different PKs groups were shown for nurse bees (I) and forager bees (J).

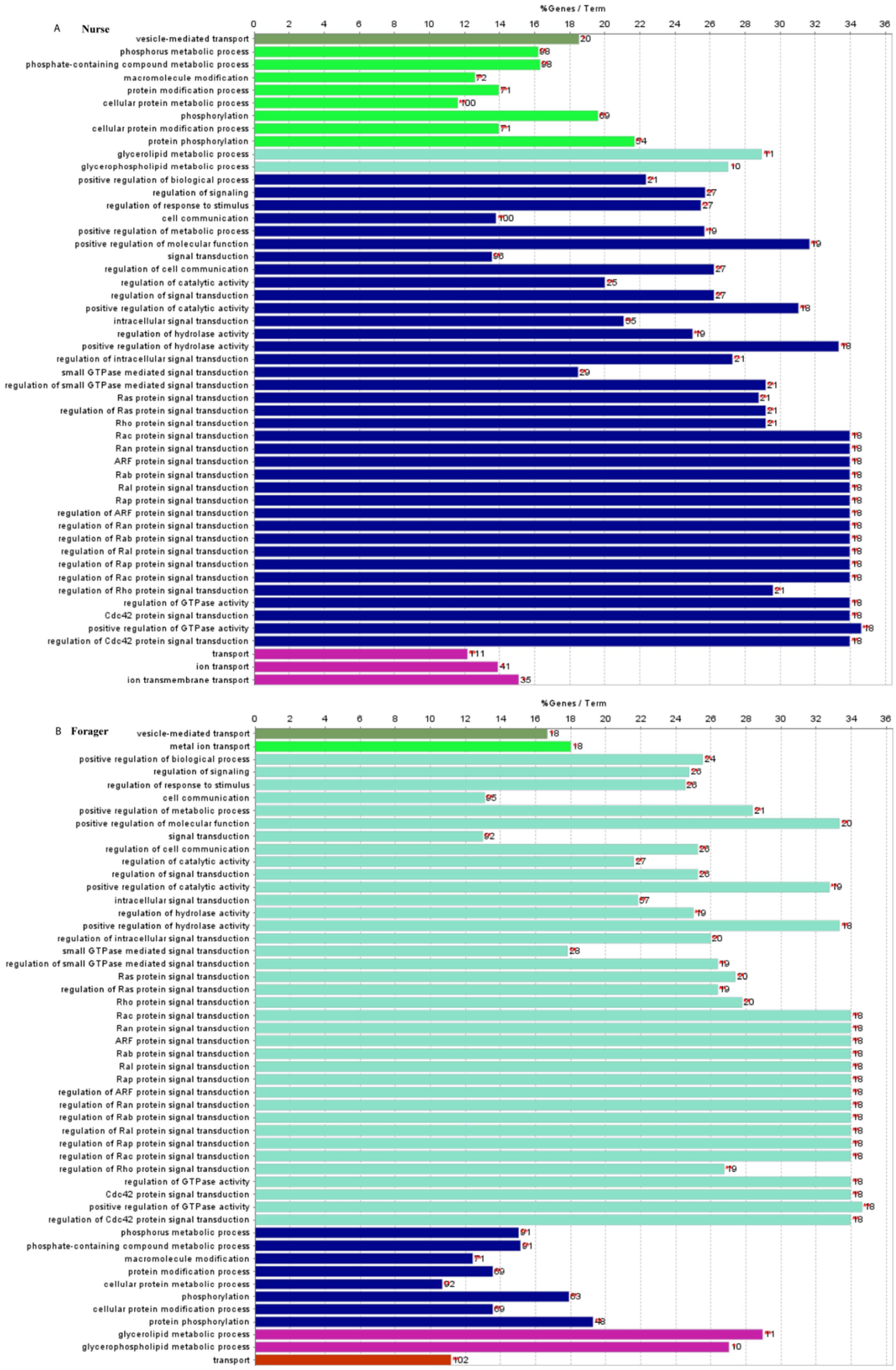

Supplemental Figure 10. Enrichment of functional GO term of the phosphoproteins identified in the brain of nurse (A) and forager (B) honeybees (*A.m.ligustica*), respectively.

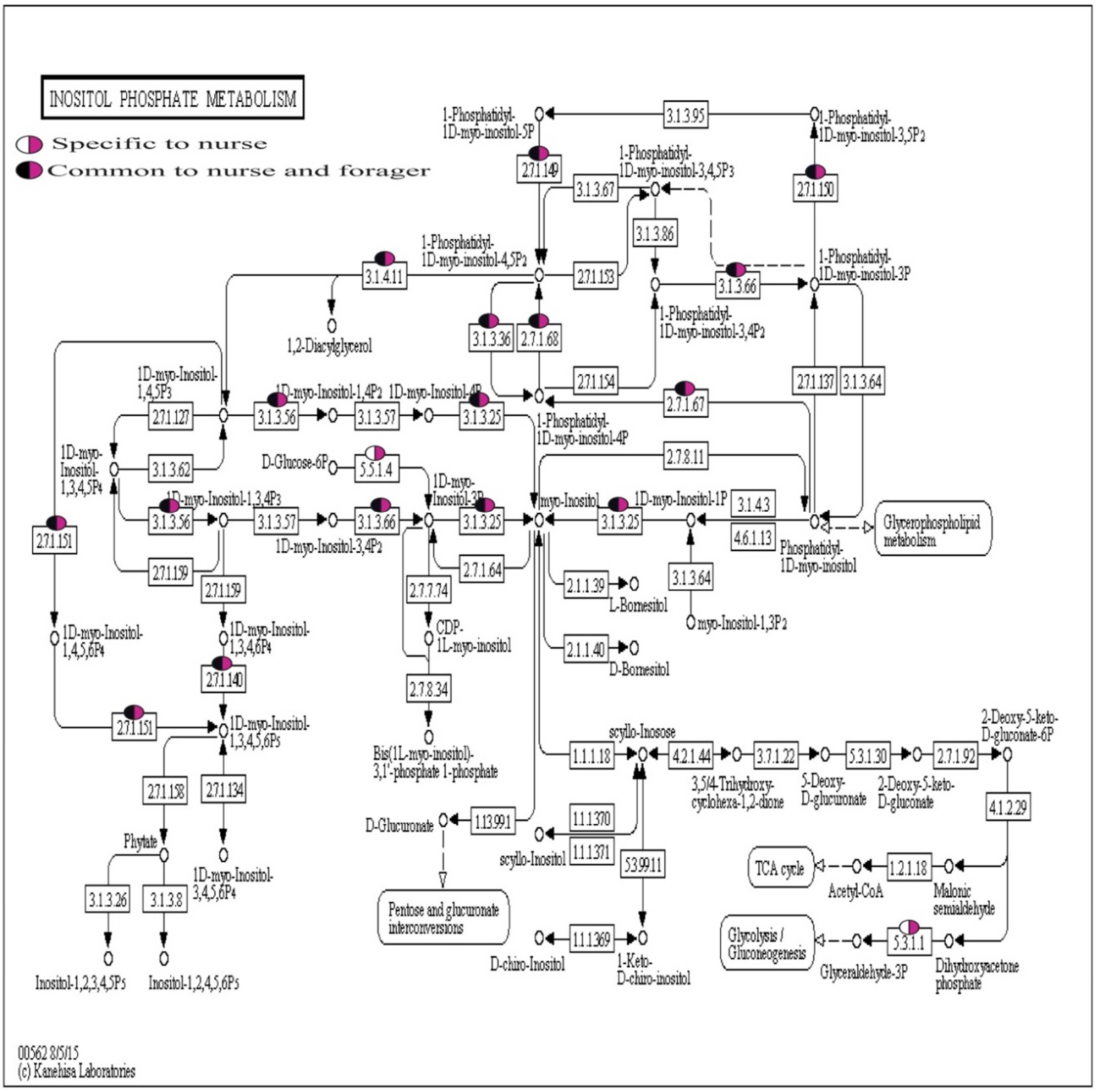

Supplemental Figure 11. The map represents a significantly enriched pathway (inositol phosphate metabolism pathway) in the phosphoprotein of the nurse and forager honeybee brains (Source of the figure [1,2])

References

1. Kanehisa, M., Furumichi, M., Tanabe, M., Sato, Y. & Morishima, and K. KEGG: new perspectives on genomes, pathways, diseases and drugs. *Nucleic Acids Res.* **45,** 1–15

(2016).

2. Kanehisa, M., Sato, Y., Kawashima, M., Furumichi, M. & Tanabe, M. KEGG as a reference resource for gene and protein annotation. *Nucleic Acids Res.* **44,** D457–D462 (2016).
